# Egyptian mandarin peel oil's anti-scabies potential via downregulation-of-inflammatory/immune-cross-talk: GC–MS and PPI network studies

**DOI:** 10.1038/s41598-023-38390-5

**Published:** 2023-08-30

**Authors:** Abeer H. Elmaidomy, Nehad M. Reda Abdel-Maqsoud, Omar. Y. Tammam, Islam M. Abdel-Rahman, Mahmoud A. Elrehany, Hussain T. Bakhsh, Faisal H. Altemani, Naseh A. Algehainy, Mubarak A. Alzubaidi, Faisal Alsenani, Ahmed M. Sayed, Usama Ramadan Abdelmohsen, Eman Maher Zahran

**Affiliations:** 1 Department of Pharmacognosy, Faculty of Pharmacy, Beni-Suef University, Beni-Suef, Egypt; 2 Department of Pathology, Faculty of Pharmacy, Deraya University, Minya, Egypt; 3 Department of Biochemistry, Faculty of Pharmacy, New Valley University, Kharga, New Valley Egypt; 4 Department of Pharmaceutical Chemistry, Faculty of Pharmacy, Deraya University, Minya, Egypt; 5 Department of Biochemistry, Faculty of Pharmacy, Deraya University, New Minya, Egypt; 6 Department of Pharmacy Practice, Faculty of Pharmacy, King Abdulaziz University, Jeddah, Saudi Arabia; 7 Department of Medical Laboratory Technology, Faculty of Applied Medical Sciences, University of Tabuk, Tabuk, Saudi Arabia; 8 Department of Biological Sciences, Faculty of Science, King Abdulaziz University, Jeddah, Saudi Arabia; 9 Department of Pharmacognosy, College of Pharmacy, Umm Al-Qura University, Mecca, Saudi Arabia; 10 Department of Pharmacognosy, Faculty of Pharmacy, Nahda University, Beni-Suef, 62513 Egypt; 11 Department of Pharmacognosy, Faculty of Pharmacy, Deraya University, Minya, Egypt

**Keywords:** Biochemistry, Drug discovery, Microbiology

## Abstract

The current study investigated the scabicidal potential of Egyptian mandarin peel oil (*Citrus reticulata* Blanco, F. Rutaceae) against sarcoptic mange-in-rabbits. Analysis of the oil's GC–MS identified a total of 20 compounds, accounting for 98.91% of all compounds found. Mandarin peel oil topical application improved all signs of infection, causing a scabicidal effect three days later, whereas in vitro application caused complete mite mortality one day later. In comparison to ivermectin, histopathological analysis showed that the epidermis' inflammatory-infiltration/hyperkeratosis-had disappeared. In addition to TIMP-1, the results of the mRNA gene expression analysis showed upregulation of I-CAM-1-and-KGF and downregulation of ILs-1, 6, 10, VEGF, MMP-9, and MCP-1. The scabies network was constructed and subjected to a comprehensive bioinformatic evaluation. TNF-, IL-1B, and IL-6, the top three hub protein-coding genes, have been identified as key therapeutic targets for scabies. From molecular docking data, compounds **15** and **16** acquired sufficient affinity towards the three screened proteins, particularly both possessing higher affinity towards the IL-6 receptor. Interestingly, it achieved a higher binding energy score than the ligand of the docked protein rather than displaying proper binding interactions like those of the ligand. Meanwhile, geraniol (**15)** showed the highest affinity towards the GST protein, suggesting its contribution to the acaricidal effect of the extract. The subsequent, MD simulations revealed that geraniol can achieve stable binding inside the binding site of both GST and IL-6. Our findings collectively revealed the scabicidal ability of mandarin peel extract for the first time, paving the way for an efficient, economical, and environmentally friendly herbal alternative for treating rabbits with *Sarcoptes mange*.

## Introduction

Sarcoptic mange (*Sarcoptes scabiei*) is a serious infectious disease that invades humans and animals all over the world^1^. The mites are highly adapted to contact with their host as contagious, burrowing, and obligate parasites. Sarcoptic mange Grower pig production is negatively impacted by adult female mites; because they mate on the skin's surface, burrow into the skin, lay eggs, and cause irritations that can lead to bleeding, reduced feeding and development, chronic stress, and decreased welfare^2,3^. The clinical picture represents chronic hyperkeratotic, which is characterized by the presence of aural crusts and many mites on the animal^4^. Similar to people, rabbits are susceptible to *Sarcoptes* infection, or mange, which reduces production and causes economic losses for rabbits, especially in the absence of effective treatment^5^. Therapy options include the systemic treatment of macrocyclic lactones, local administration of amitraz or pyrethroids, or both^6,7^. Despite their long history of effectiveness in treating mange, their extensive use has led to a decline in effectiveness because of the emergence of drug resistance. Thus, it is crucial to create novel scabicides that are both efficient and secure in order to treat and control mammalian scabies^6^.

In rabbits, goats, and pigs, several essential oils derived from *Citrus limon, Lavandula angustifolia, Citrus aurantium amara, Pelargonium asperum, Melaleuca alternifolia, Syzygium aromaticum, Eucalyptus radiata, Leptospermum scoparium, Juniperus oxycedrus, Cryptomeria japonica,* and *Cymbopogon martini*, were put to the test in real time against *S. scabiei*^8–12^. Essential oils are typically favoured over chemical acaricides since they are less harmful to animals and have a shorter environmental persistence. Also, the complex chemistry of essential oils is known to considerably impede the emergence of drug resistance against these chemicals^13^. Yet, because essential oils consist of a complex mixture of components, it might be challenging to attribute an essential oil's acaricidal properties to a specific ingredient or combination of compounds^14^. Skin irritation is yet another potential drawback that has been reported in humans^15^.

Some of the most coveted *Citrus* fruits for fresh consumption are mandarins, *C. reticulata*^16^. The more frequent name for them is "mandarin," but they are also occasionally called "tangerines." The Mandarin species includes a number of cultivars and hybrids^16^. Popularly grown varieties include *C. unshiu* Marcovitch (also known as *Unshiu mikan* in Japanese), *C. nobilis* Loureiro (also known as king mandarins), *C. deliciosa* Tenore (also known as Mediterranean mandarins), and *C. reticulata* Blanco (common mandarins)^16,17^. Mandarins are one of the main *Citrus* fruits grown in many countries such as China, Brazil, USA, India, Mexico, Spain, etc. The fruits have a great commercial worth for their essential oils and other fragrant compounds, even though they are primarily used to make pastries^18^. A lot of beverages, candies, cookies, and desserts use *Citrus* flavours^19^, while the peels of *C. reticulata* are used to flavour alcohol^19^. *Citrus reticulata* EO shown an anti-proliferative activity against rat pulmonary fibrosis produced by bleomycin (BLM) and protective properties against human embryonic lung fibroblasts (HELFs). The method is believed to involve correcting the imbalance between oxidation and antioxidation, lowering collagen deposition and fibrosis, and down-regulating lung tissue expressions of connective tissue growth factor (CTGF) and mRNA^20^. Due to its high d-limonene concentration^21^, *C. reticulata* EO demonstrated a moderate level of radical scavenging action^22^. Mandarin oil is well known for its broad spectrum antibacterial and antifungal actions. It inhibits the growth of several bacteria including *Escherichia coli, Bacillus subtilis, Pseudomonas aeruginosa, and Staphylococcus aureus*^22,23^, as well as several fungi including *Penicillium italicum, P. chrysogenum, P. digitatum, Aspergillus niger,-A. flavus, Alternaria alternata, Curvularia lunata, Rhizoctonia solani, Fusarium oxysporum, and*_*-*_*Helminthosporium oryzae*^23–26^.

**Figure 1:**
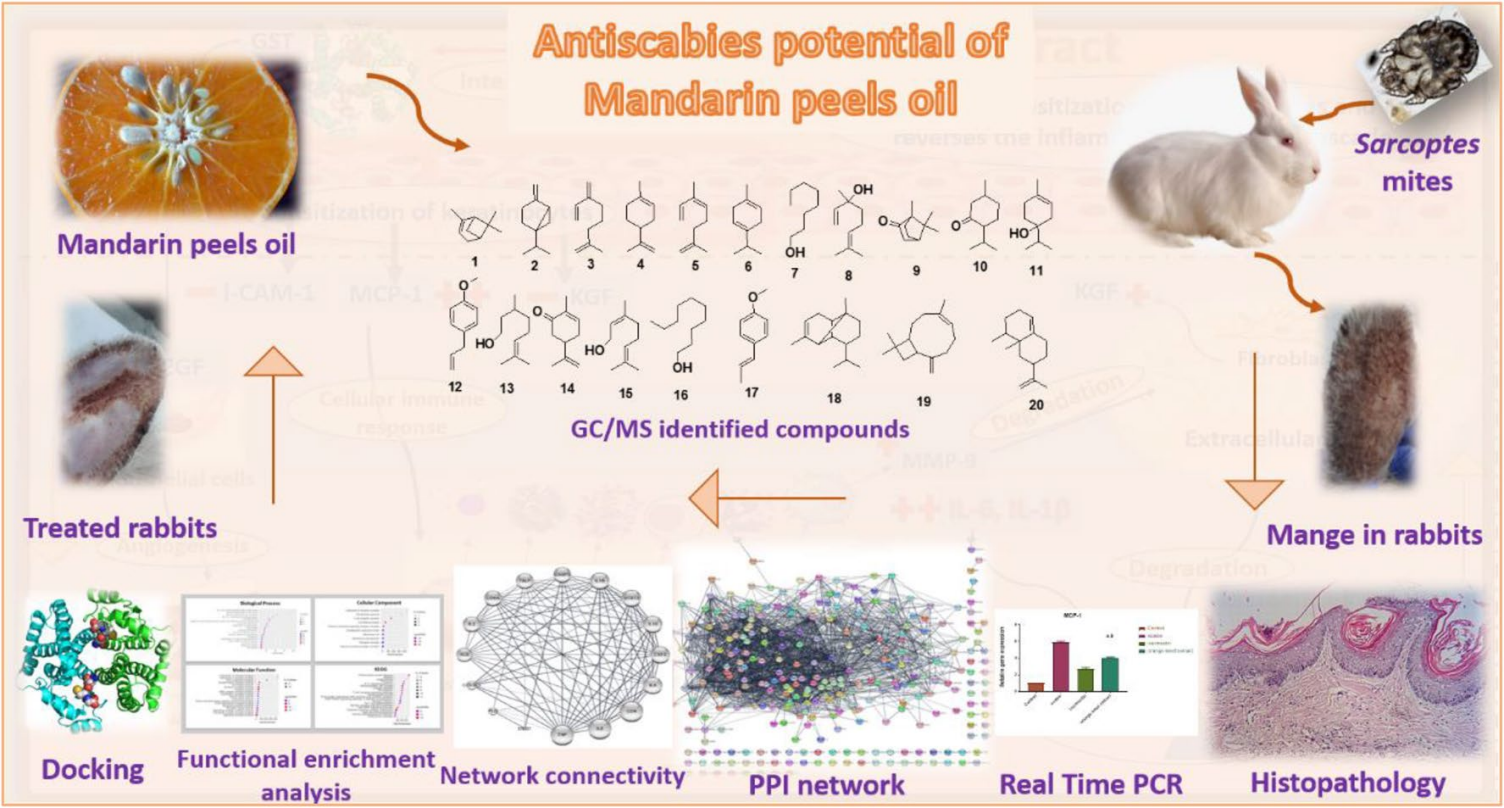
General outflow of the study.

The GC–MS profiling of mandarin peel oil has been used in the current study. Additionally, for the first time, through-in vitro*,-*in vivo,-histopathology,-mRNA-expression, and network/in silico analysis, the extract's scabicidal potential against-*Sarcoptic-mange*-in-rabbits has been investigated, allowing for the incorporation of natural candidates to proper and secure management of infectious diseases. The present investigation's framework is shown in Fig. 1.

## Material and methods

### Ethical permission

Plant materials and experiments were conducted in accordance with relevant institutional, national, and international guidelines. The study took place according to the ethical committee's permission number of 9/5/2022 at Deraya College. It was done in accordance with the National Institute of Health's guidelines for the care and use of laboratory animals and ARRIVE guidelines^27^.

### Fruit collection

In January 2021, *C. reticulata* cultivated fruits were harvested from a house garden on Atia Street in Beni-Suef, Egypt. A voucher specimen (2021-BuPD-88) was deposited at Pharmacognosy-Department, Faculty-of-Pharmacy, Beni-Suef-University, Egypt.

### Sample preparation

Using the Clevenger apparatus, the fresh peels (0.5 kg) were hydrodistillated for two hours at 75 °C. The oil was gathered, dried over anhydrous sodium sulphate, and kept in airtight amber glass vials at 4 °C for storage. On the basis of the plant material's fresh weight, the yield (v/w%) was computed^28,29^.

### GC–MS analysis

Gas chromatography-mass spectrometry (GC/MS) was used to perform chromatographic analysis on the oil recovered from peels^28,30^. The GC–MS apparatus combines a thermal mass spectrometer detector (ISQ single quadrupole mass spectrometry) with a TRACE GC ultra-high performance gas chromatograph (THERMO Scientific Corp., USA). A TR-5 MS column (30 m × 0.32 mm i.d., 0.25 mm film thickness) was installed in the GC–MS system. For the analyses, He-lium was used as the carrier gas, and the split ratio was set at 1:10 using the following temperature program: 60 C for 1 min, followed by 4.0 C/min to 240 C and a 1-min hold. At 210 °C, the injector and detector were maintained. One-liter samples of the mixes were always administered as diluted samples (1:10 hexane, v/v). By using a spectral range of m/z 40–450 and electron ionization (EI) at 70 eV, mass spectra were produced. Using AMDIS software (www.amdis.net), the chemical components of the essential oil were deconvoluted and identified by their retention indices (relative to n-alkanes C8-C22), mass spectra matching to genuine standards, and retention times (when available). (NIST Standard Reference Database, 78 Version 5.10) Wiley spectral library collection^28,31,32^.

### In vitro assays

#### In-vitro-antioxidant-activity

##### Hydrogen-peroxide-scavenging-activity

The reaction with a defined amount of exogenously provided hydrogen peroxide (H_2_O_2_) was used to determine H_2_O_2_ scavenging activity that reflects the anti-oxidative capacity of the peel oil. Colorimetric analysis was used to estimate the residual H_2_O_2_^33^. In brief, 20 µl of the sample was mixed with 500 µl of H_2_O_2_ and incubated at 37 °C for 10 min. 500 l of the enzyme/3, 5-dichloro-2-hydroxyl-benzensulfonate solution were then added, and it was incubated at 37 °C for 5 min. The colored product's intensity was quantified colorimetrically at-510-nm. A positive control was ascorbic acid. By comparing the test results to those of the control group, the percentage-of H_2_O_2_-scavenging activity-was calculated and applying the following formula:

$${\text{scavenging activity}} = \frac{{{\text{A control }}{-}{\text{A sample}}}}{{\text{A control}}} \times 100$$

IC_50_ of each sample was calculated after performing the assay at eight different concentrations : (1000, 750, 500, 375, 250, 187.5, 125 and 0 µg/mL) using Graph pad prism 7 software.

##### Superoxide radical scavenging activity

The scavenging activity of superoxide anion was measured^34^. In a Tris-HCL solution (16 mM, pH 8.0) containing 90 l of NBT (0.3 mM), 90 l of NADH (0.936 mM), 0.1 ml of peel oil (125, 250, 500, and 1000 g/mL), and 0.8 ml of Tris-HCl buffer, superoxide anion radicals were produced (16 mM, pH 8.0). After adding 0.1 ml of PMS solution (0.12 mM) to the mixture, the reaction was started. The mixture was then incubated at 25 °C for 5 min, during which time the absorbance was measured at 560 nm. Ascorbic acid was used as a model substance. Using the formula below, the percentage inhibition was calculated by comparing the test results to those of the control:

$${\text{Superoxide scavenging activity}} = \frac{{{\text{A control }}{-}{\text{A sample}}}}{{\text{A control}}} \times 100$$

IC_50_ was estimated by doing the test at four different concentrations and using the GraphPad Prism 7 software.

### Biological investigation

#### Collection-of-*Sarcoptes-scabiei*-mites

Adult-mites-were collected from rabbits that were-naturally-infected, Deraya University, Minia, Egypt's Animal House. Scraped from the borders of the lesions, the infected skin samples were then shifted-to-petri plates and-incubated within a biochemical-oxygen-demand (BOD) for an incubator for 30 min at 35 °C.

#### In vitro-application of peels oil on-*sarcoptic-mange*

A petri dish containing mites was filled with 2 ml of diluting extract (20%), along with the plates were then incubated-in-BOD. Reaction observations were made-at 1, 12, and 24 h after application. Petri plates were incubated at an ambient temperature of 25 °C and with a relative moisture of 75%, with a 5% ivermectin (1 cm3/l) group as the positive-control-and distilled water as the-negative-control. By stimulating the mites with a needle, the death of the mite was confirmed; the mite was deemed dead if it showed no response.

#### In vivo application of peels oil

The study took place on male adult rabbits for 4 weeks (weighing 2.8–3.2 kg) that were infected. The animals' ears showed clinical indicators of mange infection, such as hyperkeratinization, inflammation, redness, itching, and irritability. Microscopic mite identification in skin scrapings further corroborated this. Four groups of five rabbits each were made up of twenty animals, as follows: Five rabbits made up the normal group, the paraffin oil-positive-control-group. The-ivermectin-treated-group (5%-ivermectin). The peel-oil-group (20%-peel-oil in paraffin-oil). Paraffin oil, which is a-mineral-oil, was reportedly chosen as a diluent for the peel oil because it has little impact-on-mites^35^. Each group were kept in a separate cage, and each group received treatment by dipping the infected ears once daily. Steel hoppers were used to feed all of the rabbits, and water was available at all times. The rabbits were observed every two days to assess their clinical recovery. The goal was to find any signs of improvement in the lesions, such as the absence of irritation and redness, cutaneous smoothing, the start of the development of hair from the infection, and the cessation of scab development^10^. Skin scrapings from each rabbit's sick and healed areas were taken every three days, and Throughout the course of the therapy, they were microscopically investigated to check for sarcoptic mites with a LEICA, DM1000 microscope with a digital camera (LEICA, EC3, Germany)^10^.

#### Histopathological-examination

Tissue samples were collected at zero and three weeks following the start of the course of therapy via 20% peeling oil as well as ivermectin from healthy and infected ears. Following that, samples were dried in ethyl alcohols of increasing strength, sterilized via xylene, infused with paraffin that had been melted at 55–60 °C, and finally inserted into paraffin wax. The samples were then preserved in 10% buffered formalin. Deparaffinized, rehydrated, and stained with hematoxylin and eosin (H & E), “3–5 m thick” tissue sections were examined using-a-light-electron-microscope^36^.

#### RNA-isolation-and-qRT-PCR-assay

Using-a-digital-homogenizer-(Branson-Digital-Homogenizer®,-Danbury,-CT,-USA), 100 mg of the tissues under investigation were homogenised in 1 ml of TRIzolTM RNA Extraction Reagent (Amresco, Solon, OH, USA). RNA extraction from the biopsy sample was done in accordance with the manufacturer's instructions. RevertAid H-minus First Strand cDNA Synthesis Kits (#K1632, Thermo Science Fermentas, St. Leon-Ro, Germany) were used to create cDNA from the extracted RNA for comparable amounts of total RNA in all samples. The-qRT-PCR-was carried out on-the-Applied-Biosystems Step One Plus system using the cDNA as a template. The primers were created using the NCBI primer blast software and were produced by Invitrogen. Using the GAPDH gene as a housekeeping gene, data were analyzed using the 2CT approach^31^. Table 1 lists the primer sequences that were employed.

**Table 1:** Gene primers sequences.

Gene	Forward	Reverse
GAPDH	GTC AAG GCT GAG AAC GGG AA	ACA AGA GAG TTG GCT GGG TG
VEGF	CAT CAG CCA GGG AGT CTG TG	GAG GGA GTG AAG GAG CAA CC
IL-1	AGC TTC TCC AGA GCC ACA AC	CCT GAC TAC CTT CAC GCA CC
IL-6	GCC AAG TTC AGG AGT GAC GA	AGA GCC CAT GAA ATT CCG CA
MCP-1	GAT CCC AAT GAG TCG GCT GG	ACA GAA GTG CTT GAG GTG GTT
ICAM	GGC GGC TCA GTG TCT CAT T	TTC GTT CCC AGA GCG AGT G
IL-10	AAC AAG AGC AAG GCA GTG GA	CTA GCC GAG TTG CCA TCC TG
KGF	ACA ATG TGG CCA AAA ATG GCT	AGG AGA TTT TTC CCC TGG CG
MMP-9	GCA GAG GAG TAC CTG TTC CG	ATT ATC CAG CTC CCC CGT CT
TIMP-1	CCT TCT GCA ACT CCG ACC TT	GTA CCC GCA GAC ACT TTC CA

### In silico studies

#### Construction of protein–protein interaction (PPI) network

Using Cytoscape 3.9.1 software (https://www.cytoscape.org/)^37^ and by lunching STRING disease query tool incorporated in it which retrieves network for the top human proteins associated with the queried disease from a weekly updated web source of diseases database (https://string-db.org/)^38^ Scabies was chosen as the search term, and "Human sapiens" was chosen as the species type. The confidence score was set to 0.4, and the default settings for the remaining parameters were used to create the PPI network^39^.

#### Hub gene expression analysis

The plugin for cytoHubba the hub genes are identified using ranking techniques such as degree, edge percolated component (EPC), maximum neighbourhood component (MNC), the density of maximum neighborhood component (DMNC), and maximal clique centrality (MCC), as well as bottleneck, eccentricity, closeness, radiality, betweenness, stress, and clustering coefficient. Cytoscape is regarded as a useful exploration interface for the most significant nodes in PPI networks^40,41^.

#### Gene ontology and enrichment analysis

We employed a freely accessible bioinformatics web tool in the current investigation (ShinyGO v0.76.3). Using the many bioinformatics databases accessible, it is possible to perform both gene ontology enrichment analysis and pathway enrichment analysis. ShinyGO was used to perform the gene ontology and enrichment analysis on the 16 genes to determine the cellular elements, molecular functions, and biological processes that were impacted by this set of genes. ShinyGO retrieves comprehensive descriptions of biological signal transduction pathways from numerous databases^42^.

#### Molecular docking study

The methodologies of molecular docking intend to predict the best binding orientation of a ligand to a receptor. It proposes several suitable poses of the ligand within the active or docking site of a receptor molecule.in this study, twenty compounds that were identified underwent an in silico study by using screening for three different important-protein-targets that are heavily involved in the scabies infection process, as well as screening for potential targets at the mite itself as an acaricidal effect, in an attempt to get deep inside the mechanistic anti-scabietic effect of orange oil. The chosen targets include IL-1, which is highly effective in stimulating T cells with regulatory functions, and IL-6, which is involved in the formation of Th17 lymphocytes and the release of IL-17^43^. These cy-tokines have been identified as one of the primary molecules responsible for allergic Th2-type inflammation in the immunological response to scabies, along with TNF-, which is significant in alternative macrophage activation^44^. GSH, which is linked to the scabies defense system, takes part in a variety of processes crucial to the preservation of cells from oxygen and free radical oxidative damage^45^, Its distinctive anti-oxidant action makes it a potential target for the oil's acaricidal impact^46^. In our docking investigation, we validated the ligand and visualized the many docked poses using the computer programme MOE 2019.010. TNF- complexed with its ligand (PDB ID code: 2AZ5) is the last one, and GST is the other protein target of the mite delta class. The first protein target is (IL-1), depicted by the protein's PDB ID code of 6Y8M in co-crystallization with IL-6, as reflected by PDB ID code 1ALU, and its inhibiting ligand SX2 (a-bromo-amido-pyridine-derivative)^47^ represented by proteins (PDB ID code: 3EIN), the selected targets were acquired via the web from the Database of Proteins (http://www.rcsb.org/pdb).

### Molecular dynamic simulation

The MD simulations were carried out using NAMD 3.0.0. software^48,49^. The Charmm-36 force field is implemented in this piece of software. The protein structure was examined for missing hydrogens, the protonation states of the amino acid residues were set (pH = 7.4), and the co-crystallized water molecules were removed using the QwikMD toolkit of the VMD software. The entire assembly was then packed into a 20 solvent buffer containing 0.15 M Na + and Cl- ions in an orthorhombic box of TIP3P water. After 5 ns of equilibration, the systems were subjected to an energy minimization protocol. Force Field Toolkit (ffTK), a plugin for the VMD software, was used to determine ligand properties and topologies. After the parameters and topology files were prepared, they were imported into VMD so that the protein–ligand complexes could be read accurately, and the simulations could be run.

### Statistical analysis

The data were tabulated using the statistical programme-GraphPad-Prism-version-9 (GraphPad,-La-Jolla,-CA,-USA). To evaluate statistical differences between the groups, the ANOVA test was performed, followed by the-Bonferroni-post-hoc-test-for multiple-comparisons. The threshold for statistical significance is a p-value of 0.05 or less.

## Results

### GC–MS profiling of mandarin peels oil

**Figure 2:**
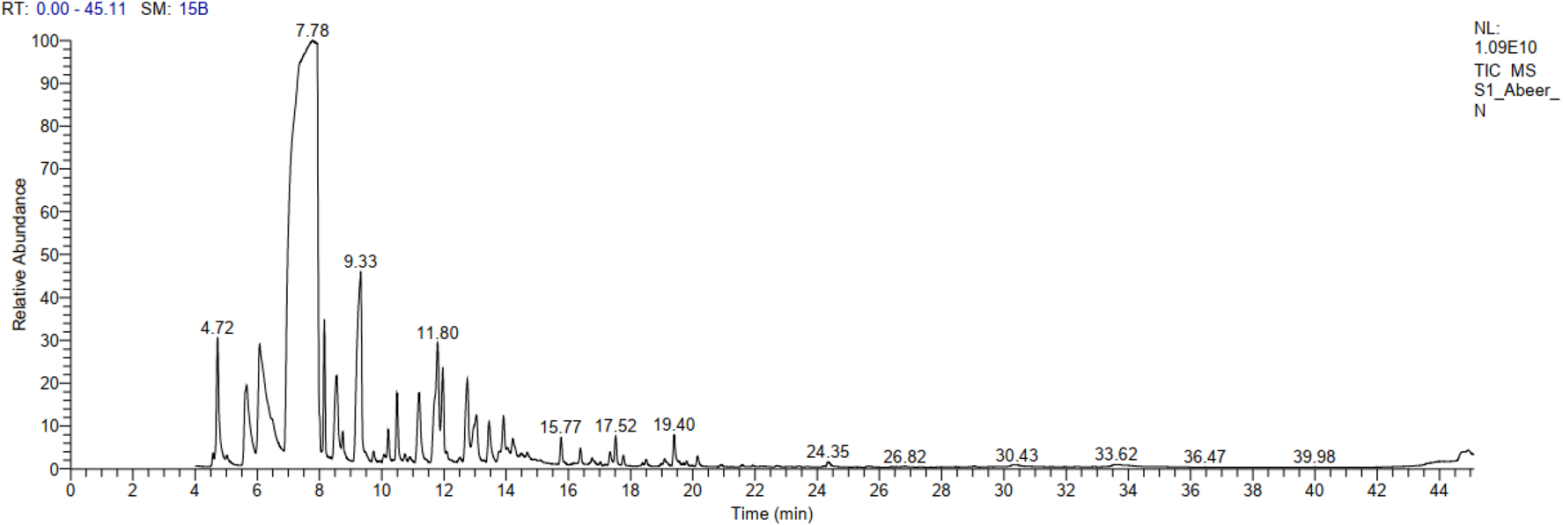
GC/MS spectrum for *Citrus reticulata* peels oil.

**Figure 3:**
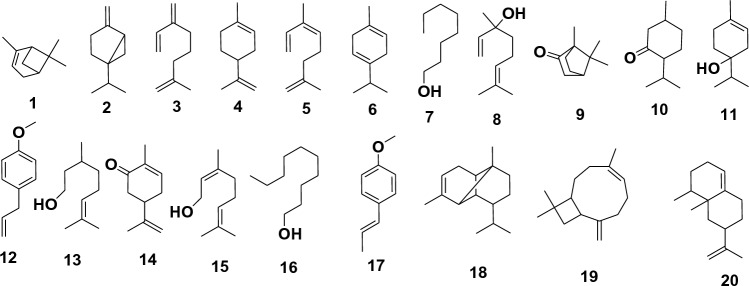
Structures of identified compounds, using GC/MS analysis, from *Citrus reticulata* oil isolated from peels.

Egyptian *C. reticulata* peels gave 2.6% v/w volatile oil fresh weight, being colourless with a characteristic odor, lighter than water, clear, transparent, and not viscous at room temperature as well as at 4 °C. GC–MS analysis was used to identify a total of 20 compounds, accounting for 98.91% of all compounds found (Table 2, Figs. 2, 3). The identified compounds **1–20** belonged to different chemical classes, including monoterpene, phenylpropene, fatty alcohol, and sesquiterpene (Table 2, Fig. 3). where monoterpenes represented 92.16% of the total identified compounds, followed by phenylpropene (3.01%), fatty alcohol (2.36%), and sesquiterpene (1.38%) (Table 2). Fourteen monoterpenes compounds (92.16%) were identified; ranging from cyclic hydrocarbon (D-limonene **4**, γ-terpinene **6**, 73.32%) which represented the major oil fraction, to oxygenated cyclic hydrocarbon ((-)-isomenthone **10**, terpinen-4-ol **11**, (-)-carvone **14**, 3.32%), and oxygenated acyclic hydrocarbon (linalool **8**, citronellol **13**, geraniol **15**, 8.78%), acyclic hydrocarbon (*α*-myrcene **3**, *α*-ocimene **5**, 2.84%), bicyclic hydrocarbon (*α*-pinene **1**, sabinene **2**, 3.45%), to oxygenated bicyclic hydrocarbon (camphor **9**, 0.45%) (Table 2, Fig. 3). Also, phenylpropene class (3.01%) contained 2.70 and 0.31% of estragole **12**, and anethole **17**, respectively. The detected fatty alcohol class contained only 1-octanol **7** and 1-decanol **16**, representing 2.36% (Table 2, Fig. 3). On the other hand, three sesquiterpene compounds (1.38%) were identified, varying from a bicyclic hydrocarbon (caryophyllene **19**, ( +)-valencene **20**, 1.00%), to a tricyclic hydrocarbon (*α*-copaene **18**, 0.38%) (Table 2, Fig. 3).

**Table 2:** *Citrus reticulata* oil composition using GC/MS analysis isolated from peels.

Nu	Identified Compound	MF	Area %	RT	RI
1	*α*-Pinene	C_10_H_16_	1.81	4.72	947
2	Sabinene	C_10_H_16_	1.64	5.60	957
3	*α*-Myrcene	C_10_H_16_	2.66	6.07	955
4	D-Limonene	C_10_H_16_	71.72*	7.50	933
5	*α*-Ocimene	C_10_H_16_	0.18	8.00	929
6	*γ-*Terpinene	C_10_H_16_	1.60	8.16	935
7	1-Octanol	C_8_H_18_O	1.71	8.54	943
8	Linalool	C_10_H_18_O	5.39	9.33	941
9	Camphor	C_10_H_16_O	0.45	10.21	912
10	(−)-Isomenthone	C_10_H_18_O	1.00	10.49	936
11	Terpinen-4-ol	C_10_H_18_O	1.34	11.20	857
12	Estragole	C_10_H_12_O	2.70	11.80	943
13	Citronellol	C_10_H_20_O	2.71	11.97	934
14	(−)-Carvone	C_10_H_14_O	0.98	13.05	927
15	Geraniol	C_10_H_18_O	0.68	13.45	937
16	1-Decanol	C_10_H_22_O	0.65	13.91	948
17	Anethole	C_10_H_12_O	0.31	14.21	910
18	*α*-Copaene	C_15_H_24_	0.38	16.39	926
19	Caryophyllene	C_15_H_24_	0.39	17.52	944
20	(+)-Valencene	C_15_H_24_	0.61	20.15	941
Total			98.91%		

*RI* Retention index relative to *n*-alkanes, *RT* Retention time (min), *MF* Molecular formula.

*Major compound, % Percentage.

According to the literature, the chemical composition of essential oils varies depending on the age of the plant, harvesting time, geographical location, and environmental conditions^50^. The Indian *C. reticulata* peels volatile oil differently from the Egyptian, having 80 compounds, where monoterpene (63.80%), represents mainly limonene (50.42%), myrecene (3.03%), and *α*-terpineol (1.19%), while sesquiterpene (12.98%) represents mainly *α*-copaene (1.49%), *β*-copaene (1.30%), and *α*-humulene (1.23%). The Indian *C. reticulata oil is* characterised by its high content of fatty acids (8.73%) and aldehyde content (7.08%), mainly *n*-hexadecanoic acid (5.65%) *α*-sinensal (3.14%)^51^. The essential oil isolated from fully matured, ripened Indian fruit peels of *C. reticulata*, on the other hand, contained 37 different components (99%). The primary ingredients included limonene (46.7%), geranial (19.0%), neral (14.5%), geranyl acetate (3.9%), geraniol (3.5%), -caryophyllene (2.6%), nerol (2.3%), neryl acetate (1.1%), and others^26^.

The essential oil constituents reported in *C. reticulata* grown in Burundi contained 58 constituents^52^. The most prevalent chemical category was monoterpene hydrocarbons (94.7%). Limonene accounted for 84.8% of the total composition, with -terpinene (5.4%), myrcene (2.2%), and -pinene (1.1%) following. Germacrene D and valencene were the primary components of the sesquiterpene hydrocarbons, which made up only 0.2% of the total composition. Compounds containing oxygen from different chemical groups made up 2.3%^52^. The two main chemical groupings were terpene alcohols (0.7%) and aliphatic aldehydes (0.7%). Linalool (0.7%), octanal (0.5%), and decanal (0.2%) made up the bulk of the mixture. In concentrations of 0.1%, octyl acetate, α-sinensal, decanol, and perillaldehyde were present. Thymol, α-sinensal, methyl thymol, as well as the acetate esters bornyl, ɣ-terpinyl, geranyl, citronellyl, and decyl acetates, were all found at concentrations of less than 0.05%^52^.

The essential oil constituents of *C. reticulata* cultivated in Algeria were reported to contain 24 constituents. Monoterpene hydrocarbons accounted for the most abundant chemical group (89.56%). The main components were limonene (67.04%), -terpinene (15.50%), and -pinene (2.75%). Sesquiterpene hydrocarbons accounted for a minor quantity (3.26%), where l-caryophyllene was the main constituent^53^.

The literature review on essential oil components in *C. reticulata* cultivated in different regions corroborates some commonalities. Consequently, limonene, a hydrocarbon monoterpene, is invariably the most common ingredient in essential oils made from *Citrus* peels, making up typically between 60 and 70 percent of the oil. However, limonene can show lower levels, as in fully matured, ripened Indian fruit peels of *C. reticulata*, in which it can decrease to 46%^26^. Also prevalent are the following substances: monoterpenes, which typically account for less than 15%, *γ*-terpinene, myrcene, and *α*-pinene, which can reach an abundance of 6.0%, 3.6%, and 1.5%, respectively.

Non-terpenoid or terpenoid compounds (aldehydes, ketones, esters, fatty acids, and phenyl) are reported to be present (1–10%) or absent according to the cultivated region, but there are no commonalities among studies reporting these compounds to have an impact on the essential oil activity or not. Sesquiterpene hydrocarbons are the most varied group of all known chemicals, and this is true for the majority of species. The most prevalent groupings also frequently include oxygenated monoterpene alcohols and monoterpene hydrocarbons.

### The antioxidant potential of mandarin peels oil

**Figure 4:**
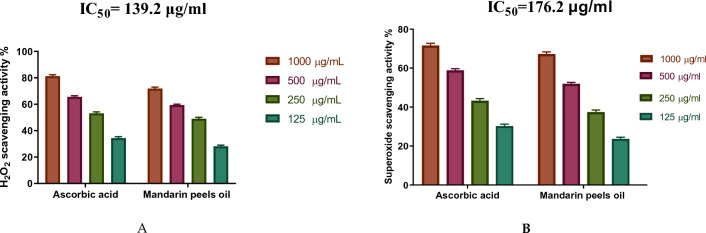
The H_2_O_2_ scavenging activity of both the mandarin peel oil and the standard increased in a concentration-dependent manner (Fig. 4**A**). Interestingly, at a concentration of 1000 μg/mL, mandarin peel oil exhibited the highest superoxide removal action, with an IC50 value of 176.2 μg/mL (Fig. 4**B**). This indicates that the oil was more effective at scavenging the superoxide radicals than the standard, ascorbic acid.

This study looked into the antioxidant activity of mandarin peel oil as-a-scavenger-potential-against-H_2_O_2_. The outcomes showed that mandarin peel oil had H_2_O_2_ scavenging capacity at a concentration of 1000 µg/mL increased in an exceedingly dose-dependent manner, compared with a standard-ascorbic-acid (IC_50_ = 139.2 µg/mL). This means that the higher the concentration of the oil, the more effectively it scavenges the H_2_O_2_ radicals (Fig. 4A).

The SOD activity of both the mandarin peel oil and the standard also increased in a concentration-dependent manner (Fig. 4B). Interestingly, at a concentration of 1000 µg/mL, mandarin peel oil exhibited the highest superoxide removal action, with an IC_50_ value of 176.2 µg/mL (Fig. 4B). This indicates that the oil was more effective at scavenging the superoxide radicals than the standard, ascorbic acid.

Overall, these findings indicate that mandarin peel oil is a potent antioxidant with a high ability to scavenge H_2_O_2_ and superoxide.

### Evaluation of the in vitro scabicidal potential of mandarin peels oil

According to in vitro data, the mandarin peel oil (20%) achieved a remarkable acaricidal impact. The mites displayed a slow movement that began at one-hour post-application-(PA) and terminated at 24 PA via 99 percent death rates, as determined by microscopic analysis.

### Evaluation of the in vivo efficacy of mandarin peels oil on infected rabbits

**Figure 5:**
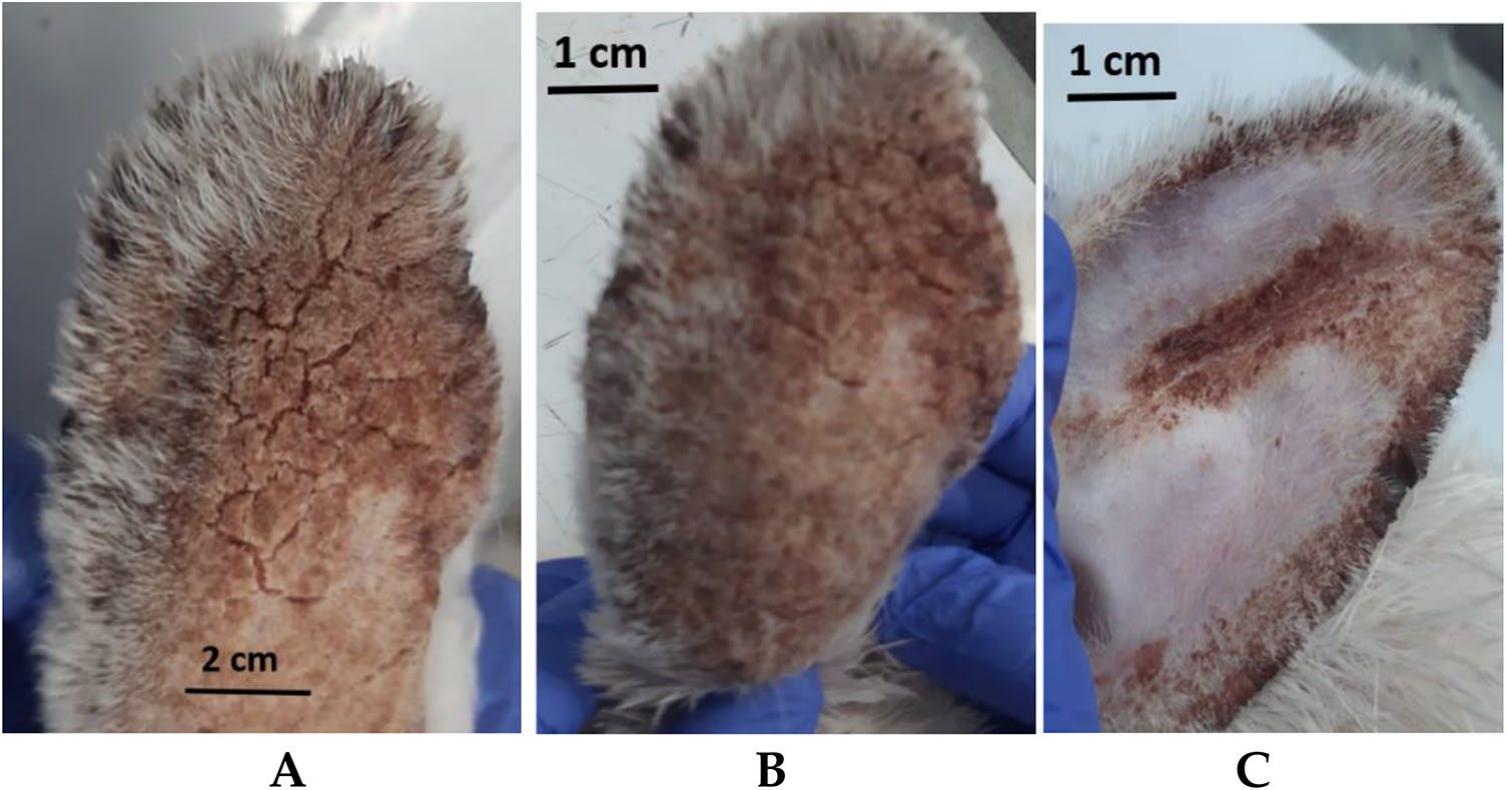
Inspection of mange-infected rabbits under a microscope, (**A**) control group (paraffin oil), (**B**) mandarin peels oil group (20% peels oil in paraffin oil), (**C**) ivermectin group (5% ivermectin)**.**

Sarcoptic mange, some chronic lesions, and scabs were visible on and inside the ears of rabbits infected with *Sarcoptes scabiei*. These animals suffered from itching, congestion, scratching, and anorexia, while those treated with mandarin peel oil (20% peel oil in paraffin oil) showed a gradual improvement in clinical symptoms from the fourth day PA through the experiment's conclusion (three weeks-PA). The lack of irritation, bleeding, scale formation, restlessness, and the appearance of smooth skin and new hair growth were signs of the recovery^54^. The ivermectin-treated animals, on the other hand, gradually improved but did not completely eradicate the condition from the seventh day PA till the investigation's conclusion (Fig. 5).

On the fifth day PA, each the peels oil along with ivermectin groups of infected animals' skin scrapings contained dead mites. By the time the animals were checked once more, on day 10, the dead mites had totally disappeared.

### Histopathological investigation

**Figure 6:**
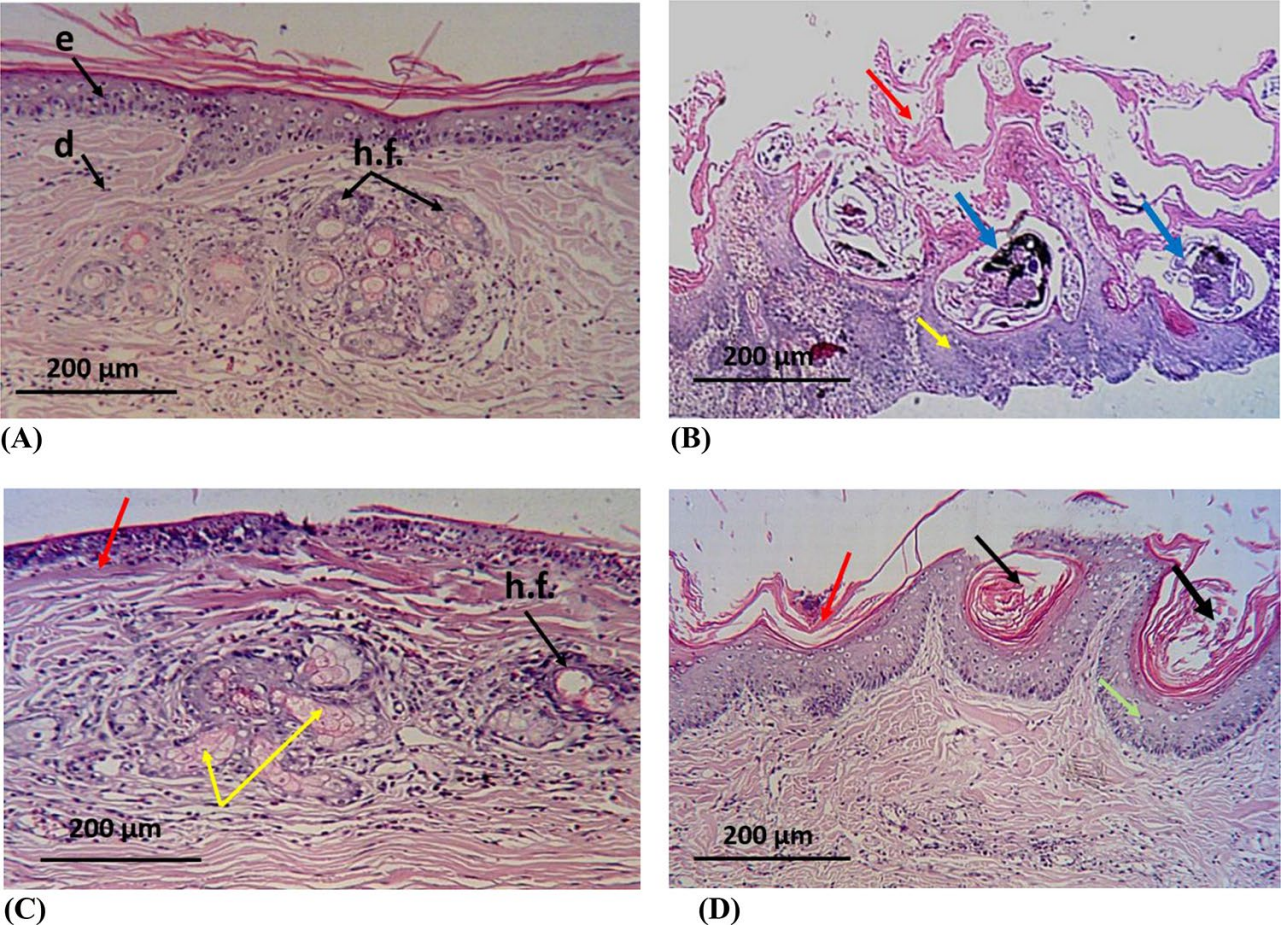
Microscopical-examination-of-skin from-different-groups of-animals, (**A**) normal-architecture-of the-skin: e; epidermis, d; dermis, h.f.; hair-follicles, (**B**) control-group-showing-skin-damage-with-hyperkeratosis (red arrows), mites-remnants-embedded-in the-skin (blue arrows), hypergranulation-of-dermis (green-arrows), severe-akanthosis-with-cellular-infiltration (black-arrows), (**C**) mandarin-peels-oil group showing-restoration of-normal-architecture, with-mild-infiltration (red-arrow), healthy-sebaceous-glands (yellow-arrow) and hair-follicles (black-arrows), (**D**) ivermectin-group showing-moderate damage-with-hyperkeratosis (red-arrow), mature-mites with-eggs-remnants-embedded in-the-dermis (black-arrow) surrounded-by-cellular-infiltration (green-arrow), and some-sebaceous-adenitis (yellow-arrows).

The normal skin's epidermis and dermis were clearly visible in the histological analyses of the normal group. The stratum corneum and stratum granulosum made up the epidermis, and the reticular layer, hair follicles, sebaceous glands, and sweat glands are visible in the dermis (Fig. 6A). Skin samples from the control group, on the other hand, displayed a changed histology, which is usual for this parasite infection^55^. Skin erosion could be seen as a result of the stratified squamous epithelial sloughing, hyperkeratosis, akanthosis, and folded, seemingly injured skin. Moreover, the epidermis, inflammatory cellular infiltration, and hypergranulating dermis all displayed necrotic debris mixed with various stages of mites (Fig. 6B).

Also, biopsy samples from the animals given mandarin peel oil (20% peel oil in paraffin oil) demonstrated a slight cellular infiltration, a lack of mites, an increase in the number of hair growth follicles, and the appearance of normal sebaceous glands are signs of progress in the skin's surface layers (Fig. 6C). The skin condition was improved in the group that had ivermectin treatment, where only a few inflammatory cells and hyperkeratosis were seen. In the outermost layer of skin, the mites' remains could be seen embedded. Cellular filtration and sebaceous adenitis were present in certain regions (Fig. 6D).

### Gene expression results

**Figure 7:**
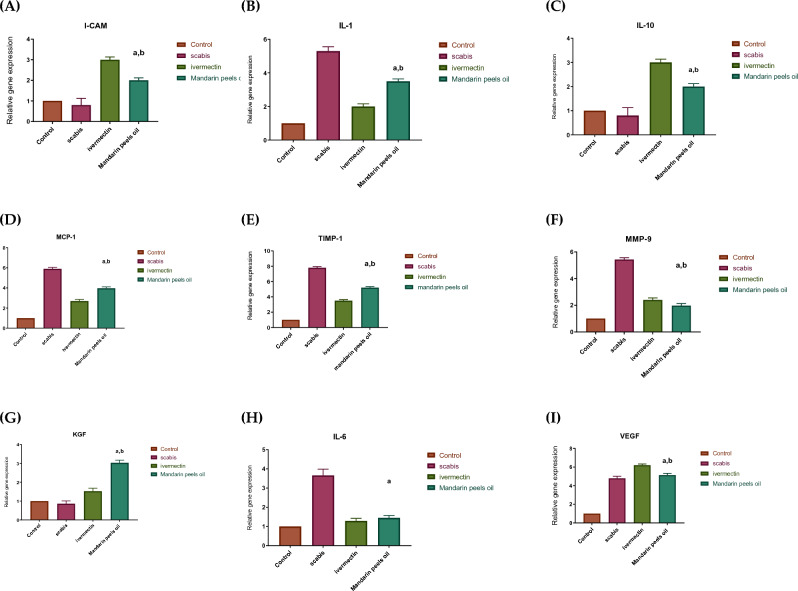
Relative gene expression in skin tissue of different animal groups using qRT-PCR After normalisation to GAPDH, (**A**) I-CAM, (**B**) IL-1, (**C**) IL-10, (**D**) MCP-1, (**E**) TIMP-1, (**F**) MMP-9, (**G**) KGF, (**H**) IL-6, and (**I**) VEGF. In comparison to the healthy control group, the data show an increase in expression by a factor of two. The mean ± SD are shown as bars. A one-way ANOVA test is used to determine whether there's a significant difference between categories, via (a) *p* < 0.05 in contrast to the normal control grouping and (b) *p* < 0.05 in contrast to the marketplace drug-induced category.

The-pro-inflammatory-cytokines-(IL-1β,-IL-6),-the-pleiotropic-cytokine-(IL-10)-and-the-monocyte chemoattractant-protein-1-(MCP-1) were all downregulated in the animals treated with mandarin peel oil (20% peel oil in paraffin oil), according to the results of q-PCR. On the other hand, 2–sevenfold increases in ICAM-1, MMP-9, VEGF, KGF, and TIMP-1 were seen (Fig. 7).

### Molecular docking study

The methodologies of molecular docking intend to predict the best binding orientation of a ligand to a receptor. It proposes several suitable poses of the ligand within the active or docking site of a receptor molecule.

#### Construction of protein–protein interaction (PPI) network

The created PPI network comprised of 296 nodes and 1725 edges, which is illustrated in Figs. S1–S3.

#### Hub gene expression analysis

**Figure 8:**
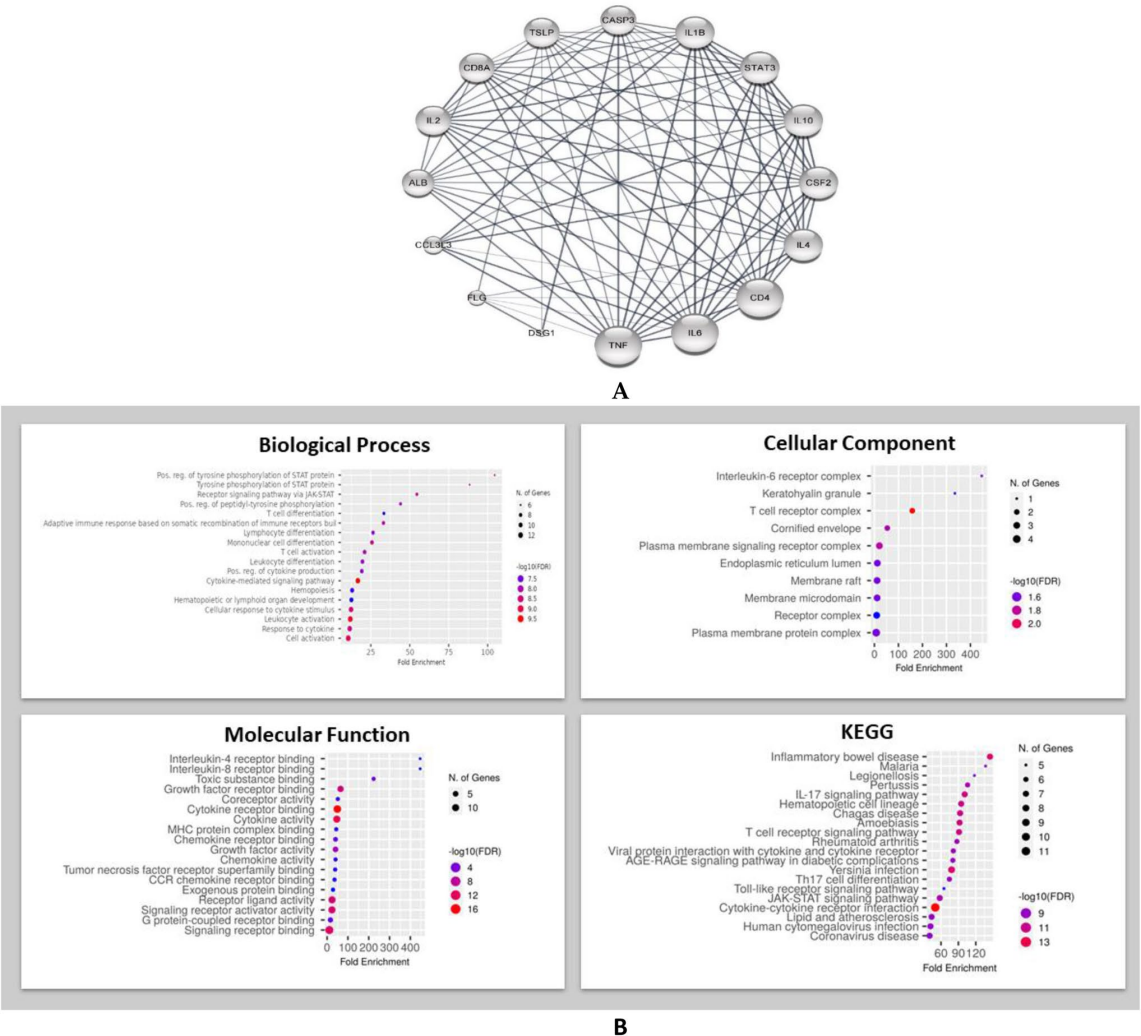
(**A**) Through a circular network design, the margins represent interactions between proteins, and the nodes serve as the hub protein criteria. Each protein's connectivity is represented by the dimension of the nodes; the larger the node, the greater its connection with other nodes in the network, (**B**) Functional enrichment analysis of filtered 16 protein coding genes by ShinyGO (https://www.genome.jp/kegg/, accessed on 12 September 2022); (http://bioinformatics.sdstate.edu/go/, accessed on 13 September 2022, a graphical gene set enrichment tool).

The *cytoHubba* plugin Cytoscape is considered a useful exploring interface for the most important nodes in the PPI networks, it used to determine the hub genes using ranking methods ,The results shown in (Table 3) demonstrated that 16 nodes repeated in more than analysis method, regarding the occurrence, IL1B possessed the highest score as it appeared in 10 methods from the 12 methods followed by Il6 and TNF-α with score of 9 for each, while CD4 appeared 8 times, IL10 and IL2 seven times (Figs. S2 and S3). In protein–protein interaction networks, it is believed that the most connected nodes (hubs) are the key players, being responsible for the most extensive pathological effects^56^ a circular layout for the filtered nodes revealed that TNF-α and IL6 possessed the highest node degree in the 16 nodes (Fig. 8A), the highest occurred protein in cytoHubba analysis IL1B with TNF-α and IL6 were chosen for in silico molecular modelling^57,58^.

**Table 3:** List of the protein coding genes present in at least two methods from twelve different methods of the *cytoHubba* plugin Cytoscape.

No	Name	Occurrence
1	IL1B	10
2	IL6	9
3	TNF-α	9
4	CD4	8
5	IL10	7
6	IL2	7
7	IL4	6
8	STAT3	5
9	CD8A	4
10	CSF2	4
11	ALB	3
12	CASP3	3
13	DSG1	3
14	TSLP	3
15	CCL3L3	2
16	FLG	2

#### Gene ontology and enrichment analysis

The Gene Ontology (GO) is considered a computational bioinformatic model of biological systems, beginning with the molecular level reaching to the organism level, GO aims to provide knowledge about the functions of gene products, namely, proteins and non-coding RNA molecules. GO is organized in three aspects. GO Molecular Functions (MF) describe activities that occur at the molecular level, Biological Processes (BP) represent the larger processes or ‘biological programs’ accomplished by multiple molecular activities and Cellular Components (CC) which are the cellular structures in which a gene product performs a function, the specific genes expressed in each cell define the identity and functionalities of that cell. Regulation of transcription is highly complex and leads to differential gene expression in specific cells or under specific conditions^59^. The analysis of the selected genes revealed that Positive regulation, and phosphorylation of STAT on JAK/STAT pathway were the top biological process in the same order while Interleukin 6 receptor complex was the top molecular component followed by keratohyalin granule and T cell receptor complex. For the Molecular function category screened genes were correlated with interleukin 4 and 8 receptor binding followed by toxic substance binding, Finally, the KEGG pathway for the selected protein coding genes were found to be involved in inflammatory bowel disease, Malaria and Legionellosis. (Fig. 8B).

#### Docking with PDB ID: 6Y8M

**Figure 9:**
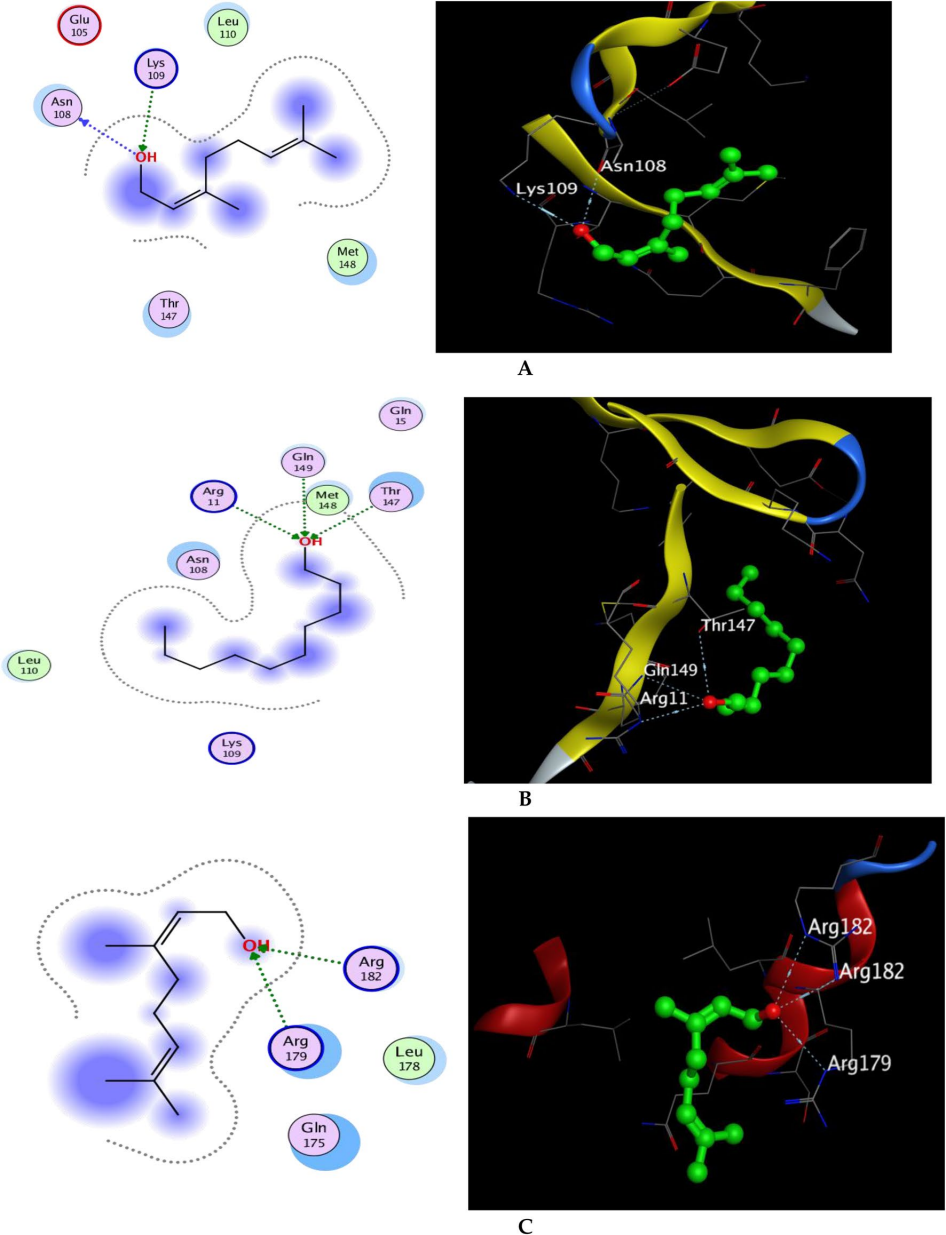

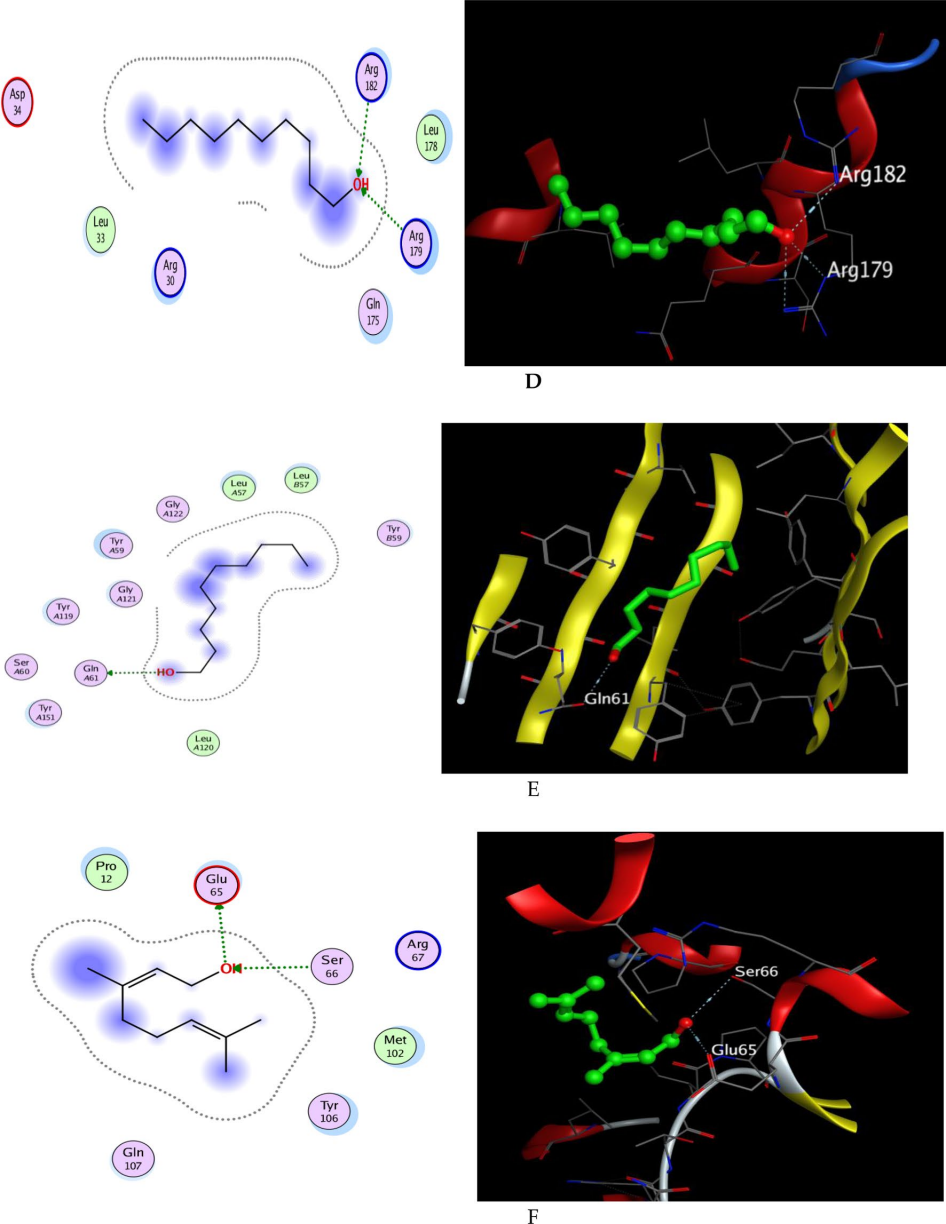
(**A**) 2D actions and 3D docking represent compound 15 (geraniol) in the successful pocket location of IL-1 (PDB: 6Y8M), (**B**) 2D actions and 3D docking represent compound 16 (1-decanol) in the successful pocket location of IL-1 (PDB: 6Y8M), (**C**) 2D actions and 3D docking present in compound 15 in the successful pocket location of IL-6 (PDB: 1ALU), (**D**) 2D relationships and 3D docking present in compound 16 in the successful pocket location of IL-6 (PDB: 1ALU), (**E**) 2D relationships and 3D docking present in compound 17 in the successful pocket location of IL-6 (PDB: 1ALU), (**E**) 2D relationships and 3D docking represent compound 16 in TNF- successful pocket location (PDB: 2AZ5), and (**F**) 2D relationships and 3D docking present compound 15 in GST successful pocket location (PDB: 2AZ5) (PDB: 3EIN).

The X-ray crystallographic structure of (IL-1β) complexed with its ligand was obtained from the Protein Data Bank (http://www.rcsb.org/pdb/,code 6Y8M). The ligand was re-docked in an active pocket at an acceptable RMSD of 1.311 and an energy score of -5.870 kcal/mol in five interactions of hydrogen bonds along with one ionic bond interaction to verify the results of our research. The involved amino acid residues in the-H-bond-interactions were Thr 147, Met 148, Gln 149, and-Arg 11 as H-acceptor, and another one with-Met-148 as-H-donor, while the ionic interaction was encountered with Arg 11. The-dock-score-of-the-twenty compounds against 6Y8M is summarized in Tables S1–S4. According to docking outcomes, compound 15 (geraniol) had a docking score of  − 5.881 kcal/mol, which was less favorable than the kinetic energy obtained by the co-crystallized ligand (Table S5), with-two-hydrogen-bond-interaction one as H-donor with Asn 108 and other as H-acceptor with Lys 109, meanwhile compound **16** (1-decanol) achieved approximately similar energy score of  − 5.625 kcal/mol when compared with the co-crystalized ligand showing three hydrogen bond interactions as H- acceptor through the hydroxyl group of 1-decanol and the amino acid residues Gln 149,Thr 147 and Arg 11 which resemble the interactions of the-co-crystalized-ligand (Fig. 9A,B).

#### Docking with PDB ID:1ALU

The X-ray crystallographic structure of IL-6 complexed with its ligand was made available by the Protein Data Bank (http://www.rcsb.org/pdb/, code 1ALU) (tartaric acid). The co-crystallized ligand (l-( +)-tartaric acid) posture was predicted by the docking method with an RMSD-of-1.758-and-an-energy-score-of  − 4.191 kcal/mol. In the style of contacts shown in Table S6, H-acceptor-interactions with Arg-182 and-Arg-179 were present, as were ionic interactions and one-hydrogen-bond-with-Gln-acting as-the-H-donor. It's interesting to note that many the 20 phytochemicals' docking results showed strong affinity for the receptor, with scores similar to the co-crystallized ligand (Table S2). It is worth mentioning that both compounds **15** and **16** exhibited better affinity towards the binding site of IL-6 than the ligand, as they showed ΔG of  − 4.372 and  − 4.401 kcal/mol respectively. The hydrogen bond interactions appeared as three hydrogen bond acceptors with amino acid residues Arg 179 and Arg 182 in both, which match the interaction pattern of the co-crystallized ligand, moreover, compound 8 also achieved a good energy score of  − 4.151 kcal/mol when compared with the ligand score of  − 4.191 kcal/mol. (Fig. 9C,D).

#### Docking with PDB ID: 2AZ5

The Protein Data Bank (http://www.rcsb.org/pdb/, code 2AZ5) provided the X-ray crystallographic structure of (TNF-) complexed with its ligand. It was demonstrated that the co-crystallized ligand was associated with 16 residues of amino acids and attached inside a small pocket, with seven of those residues coming from-chain-A and the remaining nine-from-chain-B, including-six-tyrosine-residues, from each subunit of the TNF- dimer. This inhibitor works by attaching to the cytokine's active trimer form, stimulating its dissociation into the inactive dimer form, and stabilizing it^60^. The ligand was re-docked in the active pocket to verify our research. During interactions with receptors, the ligand established hydrogen-bonds-with-Gln-61 as an H-donor-and-with-Tyr-119 as a pi-H interaction. The co-crystallized ligand pose was predicted by the docking method with the least RMSD and an energy score of  − 6.923 kcal/mol. Figure 9E, Table S7. Table S3 summarizes the dock scores of the 20 compounds against 2AZ5. Due to its hydroxyl moiety and the amino acid sequence Gln 61, compound 16 was the only one to obtain a dock score of  − 5.129 kcal/mol with a single hydrogen bond interaction as an H-donor (Fig. 9).

#### Docking with PDB ID:3EIN.

The-Protein-Data-Bank-(http://www.rcsb.org/pdb/,code 3EIN) has-the-X-ray-crystallographic-structure-of-Drosophila melanogaster's delta-class-GST. When glutathione was redocked, it revealed four hydrogen bond interactions, two of which included Arg-67-and-Ser-66 as H-acceptors and the-other-two-involving Glu 65-and Ile 53-as H-donors. The ligand's energy score was  − 5.945 kcal/mol in addition to the two ionic interactions with Arg 67 and Glu 65 (Table S8). based on the investigated compounds' docking results reported in Table S4, compound 15 (geraniol), which displayed a G of  − 5.861 kcal/mol, exhibits strong similarities to glutathione in terms of energy score. In a similar manner to the co-crystallized ligand, geraniol interacted with the binding site of the GST receptor by forming two hydrogen bonds, one with the amino acid residue Ser-66 as an H-acceptor-and the-other with-Glu 65 as an-H-donor (Fig. 9F).

#### Molecular dynamics simulation

**Figure 10:**
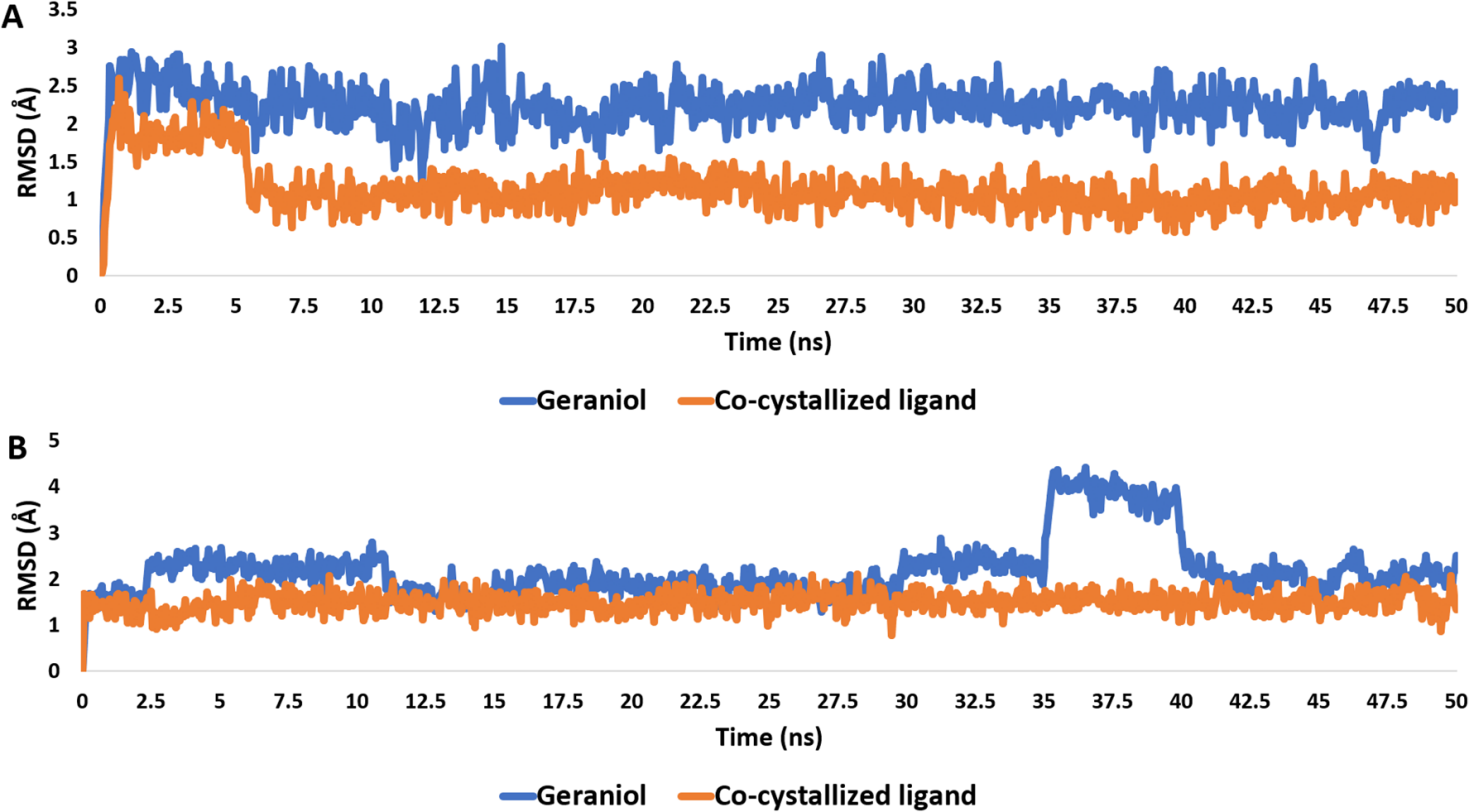
RMSDs of geraniol inside both GST and IL-6 in comparison with the co-crystalized ligand of each protein [(**A**) and (**B**) respectively] over the course of 50 ns-long MD simulation.

To validate the docking outcomes, the best-scoring docking pose of geraniol with both GST and IL-6 were subjected to 50 ns-long MD simulation. As shown in Fig. 10, geraniol modelled structure achieved stable binding inside the binding site of each protein with RMSD profiles (~ 2.5 Å) comparable with that of the co-crystallized ligands (~ 1.7 Å). These results suggest geraniol as a probable inhibitor of both GST and IL-6.

#### In silico druglikeness of compounds 15 and 16.

**Figure 11:**
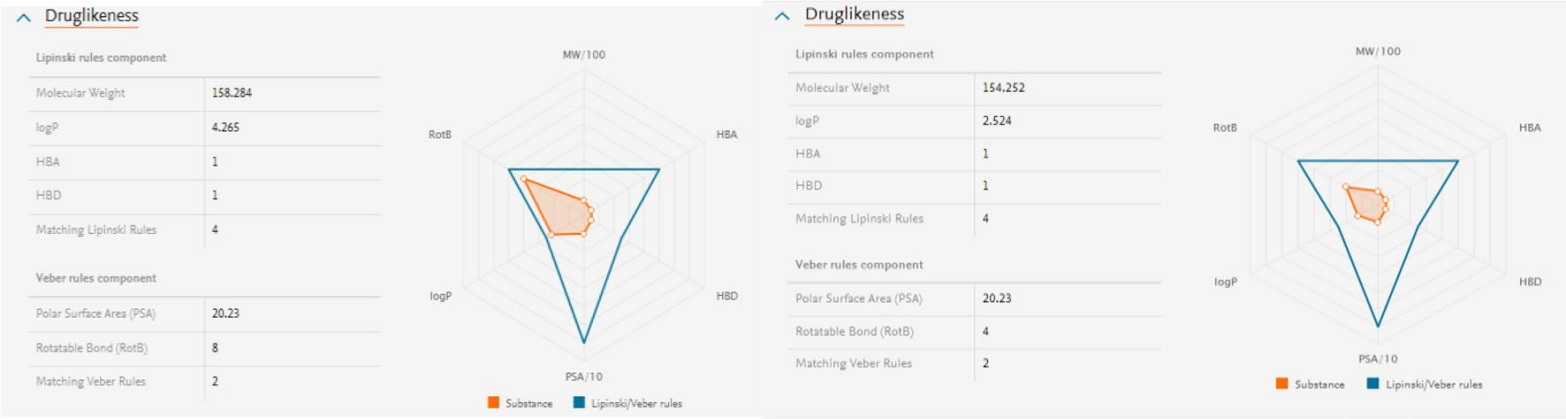
In silico druglikeness (Lipinski and Veber rules) of compounds **15** and **16**.

Various physicochemical properties of a given drug may have a significant impact on its bioactivity, as they are closely related to interactions between the drug and its potentially suspected target. Recently, in silico approaches introduced a powerful tool for drug discovery to assess the proposed pharmacokinetics (ADME) of compounds, which play a vital role in their pharmacological activities, especially at the early stages of screening for lead compounds. Consequently, the measurement of these parameters is of great value in the selection of an efficient drug candidate. Lipinski and Veber’s rules are successful tools to perform such screening, as Lipinski’s rule of five states that a compound has drug-like activity if at least three of the following criteria are achieved: a molecular mass less than 500 Da, a maximum of five hydrogen donors, a maximum of 10 hydrogen bond acceptors, and a partition coefficient between octanol and water (LogP (o/w)) smaller than 5. According to Veber’s rule, a compound is orally active if it has 10 or fewer rotatable bonds and a polar surface area (PSA) greater than 140 Å. For predicting drug-like properties, we used Reaxys. The screening of compounds **15** and **16** revealed that all of them complied with Lipinski and Veber’s rules (Fig. 11).

## Discussion

Essential oils (EOs) are generally well tolerated, as evidenced by their widespread use in food, hair, and skin preparations^61^. In comparison to conventional drugs, EOs are less likely to cause resistance due to their multiple active components^61^. EOs may have antibacterial, anti-inflammatory, and antipruritic properties in addition to their scabicide properties^8,14^. All these adjuvant properties are especially appealing for the treatment of scabies.

As a result of mites burrowing deeply into the skin, scabies pathogenesis is complicated and involves a number of mechanisms, including parasite persistence, which has an impact on both the structure and function of skin^62^. All these elements work together to make treatment ineffective, especially given that most synthetic medications kill mites rather than altering the immune system or promoting tissue repair. In light of this, plant-derived phytochemicals can operate as safe substitutes for synthetic options for the eradication of infectious diseases due to their broad therapeutic potential and negligible adverse effects^63^. *Citrus* fruits have reportedly been found to have immunostimulatory, anti-inflammatory, antimicrobial, and antioxidant properties^64–66^. It had considerable antibacterial efficacy against *S. aureus* and Candida-skin-infections, including-oral-and-vaginal-candidiasis^65,67–70^. Fascinating studies have shown that *Citrus* oil may change the way that inflammatory responses are expressed, suppressing pro-inflammatory cytokines and enhancing skin's defensive barriers^71^. No investigation has yet been conducted, to the best of the information we have, on the acaricidal-potential-of mandarin peel oil against *Sarcoptes scabiei*.

Therefore, the current study examined the GC/MS makeup of mandarin peel oil and assessed the oil's ability to kill Scabies-mites-in-both in vitro-and in vivo testing. There were no symptoms of skin irritability, inflammation, or unease during or after the application of mandarin peel oil. Our findings showed that orange peel oil could have a substantial acaricidal effect on *Sarcoptes scabiei* mites 24 h after application. The animals' skin began to exhibit healthy symptoms after the mites died, including the cessation of inflammation-and-hyperkeratosis, the emergence of new-skin-layers, and-the beginning of new hair growth. This occurred at the same time as reports of the effective treatment of rabbit mange^72^. This full recovery was seen after 3 weeks, whereas the ivermectin group's healing continued until the completion of the experiment (4 weeks) without leading to full recovery. The histopathological findings revealed that the dermis and epidermis of the treated animals improved, inflammation cells decreased, and mite remains were not present in the skin layers. The death of mites, as well as the absence of inflammation, pruritis, skin damage, and scale formation, are the primary causes of the improvement^55^. In contrast, the skin layers of the ivermectin group saw gradual alterations throughout therapy, and at the end of the investigation, some inflammatory cells as well as traces of deceased mites were still visible. This could be explained by the potent anti-mite effects of ivermectin as well as the common itching and allergic reactions brought on by topical deltamethrin use, which extends inflammation and causes additional delays the emergence of good indicators^73^.

Epidermal-keratinocytes-as the-first line-of defense against hazardous external invaders must be used to obtain understanding of the-modulative-effects-of mandarin peel oil on the-pathophysiology-of scabies. To recognize various infections and launch immune responses, keratinocytes generate recognition receptors on their surfaces. These receptors allow them to secrete cytokines, chemokines, and anti-microbial peptides that help attract inflammatory cells^74^. Any imbalance in the activity of keratinocytes, which is crucial for the control of skin immunological homeostasis, might lead to illness. Our results show that when exposed to live digging scabies mites or their waste products (including saliva or eggs as well), a significant amount of genes in the skin fibroblasts and keratinocytes change their expression, which further activates other cell types^75^. Hence, in response to scabies, many other skins cell categories, such as lymphoid cells, endothelial cells, or LCs, and dendritic cells, have complex interactions (cross talk), which results in the development of inflammatory and oxidative stress states. This could increase reactive oxygen species like H_2_O_2_, which leads to lipid peroxidation and negatively affects the skin's structure and permeability. In our investigation, faster clinical and parasitological recovery confirmed the potential antioxidant action of mandarin peel oil by restoring the altered oxidant/antioxidant balance in treated animals to normal. Antioxidants are believed to hasten wound healing by reducing oxidative stress on the wound. They are essential in preventing harm from being done to biological elements like DNA, proteins, lipids, and bodily tissue when reactive species are present^31^. Because the elevated levels of ROS at the site of injury are the main promoters of collagen disintegration, the breakdown of the extracellular matrix (ECM), a decrease in vascular development and re-epithelialization, and a rise in cytokines that are pro-inflammatory, all of which extend inflammation, an extract with ROS scavenging potential could be a key component of the healing protocol^31^.

**Figure 12:**
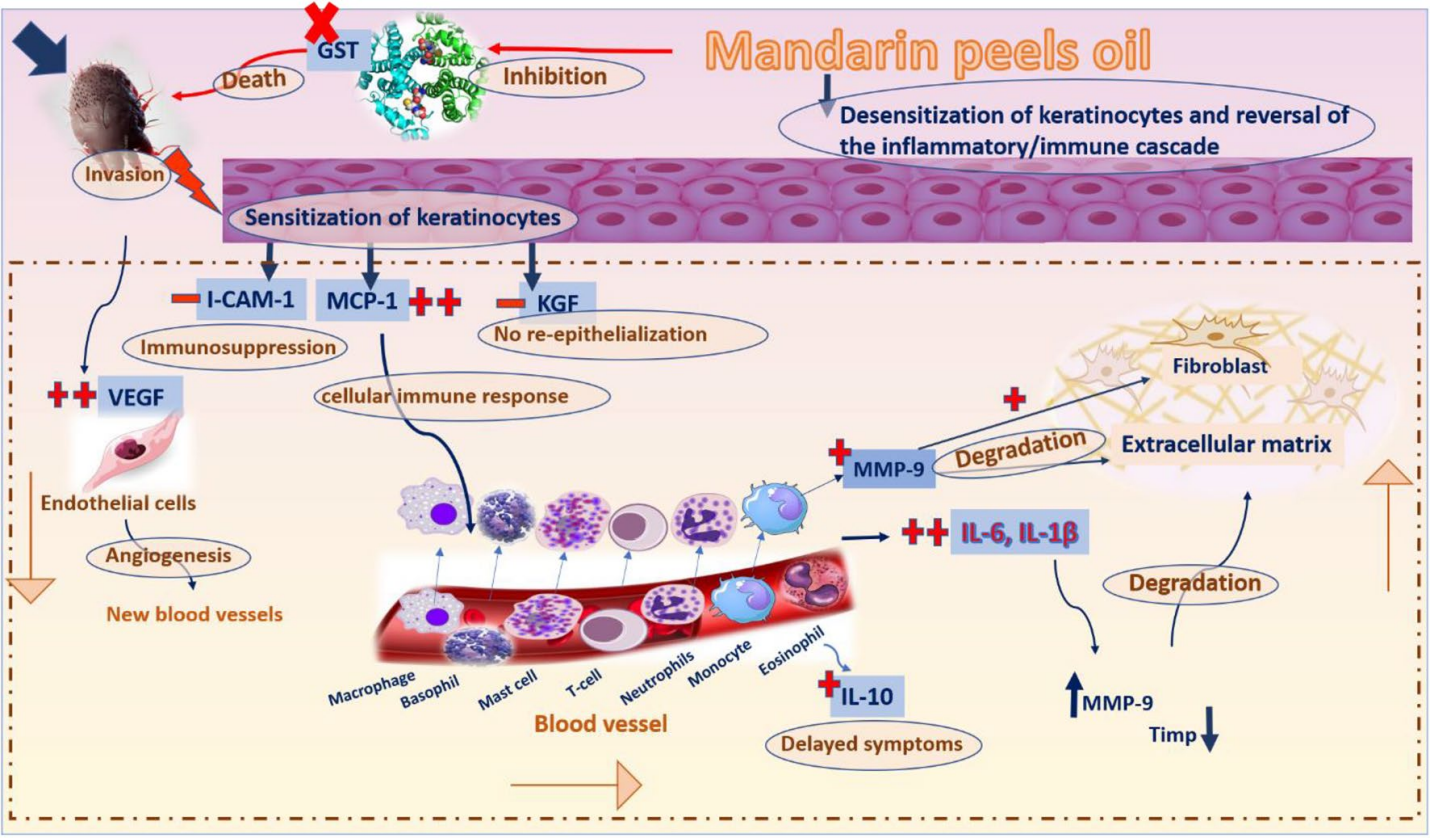
The suggested mechanism for the effect of mandarin peel oil on scabies-infected rabbits.

The anticipated mechanism of action of mandarin peel oil on scabies-infected rabbits was shown schematically in Fig. 12. Infiltrating mites, based on reports, activate the skin's keratinocytes, and exhibit a capacity to suppress the immune system's response by lowering the expression of genes of i-CAM-1, an intracellular adhesion molecular structure noticed on endothelial surface cells. This decreases the blood-supply and-immune cells to the penetration site and lessens the protective abilities of both lymphocytes and neutrophils. On the other hand, an infection increases MCP-1, a chemokine that stimulates immune cells and causes inflammation^76^. Clinical signs are not noticed for 4–6 weeks after an individual has been diagnosed with scabies mites. This is since the regulatory T cells (type 1) are induced to produce IL-10, a cytokine with anti-inflammatory properties that is required by humans to prevent inflammatory and autoimmune illnesses^77^. Furthermore, mite products that sensitize keratinocytes are likely to raise the production of VEGF, which is additionally induced by the mites to raise angiogenesis. The mites raise the flow of blood in the region in order to get the nourishment they require from the food being consumed, which worsens inflammation^77^. A delayed re-epithelialization of the wound is the result of decreased KGF receptor signaling, which also lowers the rate of proliferation of epidermal keratinocytes along the wound edge. Matrix metalloproteinase (MMP-9) is one of a set of hydrolase enzymes that are expressed in many severe conditions, including wounds, osteoarthritis, ischemia, and viral disorders. The inflammation also significantly increases MMP-9 levels^78^. Nearly all parasite infections use MMP-9 to remodel tissue, which often slows down the production of ECM molecules including collagen II and aggrecan^79^. TIMP-1 (tissue inhibitor of met-alloproteinase) tightly regulates the biological activities of MMPs, and proteolysis results from an imbalance in the MMPs/TIMPs ratio^80^. Reversing the activation of this network of interrelated genes may therefore be a useful treatment approach to slow the spread of scabies. When CSE was applied topically, the expression of IL-1, 6, 10, VEGF, MMP-9, and MCP-1 significantly decreased, whereas the expression of i-CAM-1, KGF, and TIMP-1 significantly increased.

The outcome was an improvement in host immunity against invading mites, a decrease in pro-inflammatory cytokines and an increase in anti-inflammatory ones, which could reverse the unfavorable symptoms and lead to improved re-epithelialization, rapid recovery, and a decrease in inflammation.

## Conclusion

With instances of treatment failure and the emergence of resistance, controlling scabies effectively using the available acaricidal medicines has proven to be extremely difficult. With a biocidal performance comparable to that of traditional synthetic treatments, this study demonstrated the mandarin peel oil's acaricidal efficacy against mange mites in rabbits. The work examined the composition of the oil and revealed the presence of different hydrocarbons and their oxygenated forms, with proved biocidal activities. Additionally, the oil has been tested against naturally infected rabbits with mange using different techniques and proved higher efficacy and safety compared to market agents. The mandarin peel oil presents an ideal alternative to commercial medications used for the control of arachnids that can harm humans and animals while being economical, safe, and environmentally friendly. These candidates can be successfully employed to create novel biocides for applications in agricultural improvement and livestock protection.

### Supplementary Information

Supplementary Information 1.

Supplementary Information 2.

## Data Availability

All data generated or analyzed during this study are included in this article (and its supplementary information files).
