# Supplementary material for: Egyptian mandarin peel oil's anti-scabies potential via downregulation-of-inflammatory/immune-cross-talk: GC–MS and PPI network studies
Source: Sci Rep. 2023 Aug 30;13:14192. doi: 10.1038/s41598-023-38390-5 (PMC10469216; doi:10.1038/s41598-023-38390-5)
Supplement: Supplementary file 2 — Supplementary Information 2. [file 41598_2023_38390_MOESM2_ESM.docx]

SUPPLEMENTARY DATA

**Egyptian Mandarin Peel Oil's Anti-Scabies Potential Via Downregulation-of-Inflammatory/Immune-Cross-Talk: GC-MS and PPI Network Studies**

**Abeer H. Elmaidomy^1*^, Nehad M. Reda Abdel-Maqsoud^2^, Omar. Y. Tammam^3^, Islam M. Abdel-Rahman^4^, Mahmoud A. Elrehany^5^, Hussain T. Bakhsh^6^, Faisal H. Altemani^7^, Naseh A. Algehainy^7^, Mubarak A. Alzubaidi^8^, Faisal Alsenani^9^, Ahmed M. Sayed^10^, Usama Ramadan Abdelmohsen ^11^* and Eman Maher Zahran^11^**

^1^Department of Pharmacognosy, Faculty of Pharmacy, Beni-Suef University, Beni-Suef, Egypt. Abeer011150@pharm.bsu.edu.eg

^2^Department of Pathology, Faculty of Pharmacy, Deraya University, Minia, Egypt. nehad.reda@deraya.edu.eg

^3^Department of Biochemistry, Faculty of Pharmacy, New valley University, New valley, Egypt. omar.tammam93@pha.nvu.edu.eg

^4^Department of Pharmaceutical Chemistry, Faculty of Pharmacy, Deraya University, Minia, Egypt. dr.islam_moh@deraya.edu.eg

^5^Department of Biochemistry, Faculty of Pharmacy, Deraya University, New Minia, Egypt. mahmoud.elrehany@deraya.edu.eg

^6^Department of Pharmacy Practice, Faculty of Pharmacy, King Abdulaziz University, Jeddah, Saudi Arabia. htbakhsh@kau.edu.sa

^7^Department of Medical Laboratory Technology, Faculty of Applied Medical Sciences, University of Tabuk, Tabuk, Saudi Arabia. faltemani@ut.edu.sa, nalgehainy@ut.edu.sa

^8^Department of Biological Sciences, Faculty of Science, King Abdulaziz University, Jeddah, Saudi Arabia; mahalzubaidi@kau.edu.sa

^9^Department of Pharmacognosy, College of Pharmacy, Umm Al-Qura University, Makkah, Saudi Arabia; [fssenani@uqu.edu.sa](mailto:fssenani@uqu.edu.sa)

^10^Department of Pharmacognosy, Faculty of Pharmacy, Nahda University, 62513 Beni-Suef, Egypt.

^11^Department of Pharmacognosy, Faculty of Pharmacy, Deraya University, Minia, Egypt. usama.ramadan@mu.edu.eg, eman.maher@deraya.edu.eg

***** Correspondence: usama.ramadan@mu.edu.eg (U.R.A.), Abeer011150@pharm.bsu.edu.eg (A.H.E.).

**TableS1.** Binding energies and RMSD of the 20 Compounds and ligand into the active pocket site of catalytic domain of IL-1β (PDB: 6Y8M).

| **NO** | **Compound** | **S_a_ kcal/mole** | **RMSD_Refine_b_** |
| --- | --- | --- | --- |
| **1** | **α-Pinene** | **-3.697** | **1.382** |
| **2** | **Sabinene** | **-3.757** | **1.851** |
| **3** | **α-Myrcene** | **-3.716** | **1.35** |
| **4** | **D-Limonene** | **-3.589** | **1.911** |
| **5** | **α-Ocimene** | **-3.682** | **1.798** |
| **6** | **γ-Terpinene** | **-3.512** | **1.814** |
| **7** | **1-Octanol** | **-3.663** | **1.423** |
| **8** | **Linalool** | **-4.236** | **1.068** |
| **9** | **Camphor** | **-3.569** | **1.454** |
| **10** | **(-)-Isomenthone** | **-3.751** | **1.61** |
| **11** | **Terpinen-4-ol** | **-3.869** | **1.376** |
| **12** | **Estragole** | **-3.645** | **1.694** |
| **13** | **Citronellol** | **-3.912** | **1.372** |
| **14** | **(-)-Carvone** | **-3.68** | **1.793** |
| **15** | **Geraniol** | **-5.881** | **1.653** |
| **16** | **1-Decanol** | **-5.625** | **1.047** |
| **17** | **Anethole** | **-3.615** | **1.912** |
| **18** | **α-Copaene** | **-3.993** | **1.807** |
| **19** | **Caryophyllene** | **-3.872** | **1.66** |
| **20** | **(+)-Valencene** | **-4.13** | **1.077** |
| **#** | **ligand** | **-5.87** | **1.311** |

**TableS2.** Binding energies and RMSD of the 20 Compounds and ligand into the active pocket site of catalytic domain of IL-6 (PDB: 1ALU).

| **NO** | **Compound** | **S_a_ kcal/mole** | **RMSD_Refine_b_** |
| --- | --- | --- | --- |
| **1** | **α-Pinene** | **-3.509** | **1.098** |
| **2** | **Sabinene** | **-3.437** | **1.31** |
| **3** | **α-Myrcene** | **-3.839** | **1.419** |
| **4** | **D-Limonene** | **-3.612** | **1.132** |
| **5** | **α-Ocimene** | **-3.619** | **0.753** |
| **6** | **γ-Terpinene** | **-3.428** | **1.106** |
| **7** | **1-Octanol** | **-3.997** | **1.263** |
| **8** | **Linalool** | **-4.151** | **1.801** |
| **9** | **Camphor** | **-3.507** | **1.193** |
| **10** | **(-)-Isomenthone** | **-3.664** | **1.82** |
| **11** | **Terpinen-4-ol** | **-3.662** | **0.911** |
| **12** | **Estragole** | **-3.85** | **1.445** |
| **13** | **Citronellol** | **-3.907** | **1.607** |
| **14** | **(-)-Carvone** | **-3.672** | **1.487** |
| **15** | **Geraniol** | **-4.372** | **1.543** |
| **16** | **1-Decanol** | **-4.401** | **1.106** |
| **17** | **Anethole** | **-3.389** | **1.794** |
| **18** | **α-Copaene** | **-3.948** | **1.467** |
| **19** | **Caryophyllene** | **-4.015** | **1.268** |
| **20** | **(+)-Valencene** | **-3.839** | **1.534** |
| **#** | **ligand** | **-4.191** | **1.758** |

**TableS3.** Binding energies and RMSD of the 20 Compounds and ligand into the active pocket site of catalytic domain of TNF-α (PDB: 2AZ5).

| **NO** | **Compound** | **S_a_ kcal/mole** | **RMSD_Refine_b_** |
| --- | --- | --- | --- |
| **1** | **α-Pinene** | **-4.142** | **0.845** |
| **2** | **Sabinene** | **-4.57** | **0.714** |
| **3** | **α-Myrcene** | **-4.609** | **1.414** |
| **4** | **D-Limonene** | **-4.147** | **1.117** |
| **5** | **α-Ocimene** | **-4.636** | **1.174** |
| **6** | **γ-Terpinene** | **-4.1** | **1.424** |
| **7** | **1-Octanol** | **-4.332** | **0.906** |
| **8** | **Linalool** | **-4.607** | **0.975** |
| **9** | **Camphor** | **-4.009** | **1.419** |
| **10** | **(-)-Isomenthone** | **-4.12** | **1.745** |
| **11** | **Terpinen-4-ol** | **-4.235** | **1.202** |
| **12** | **Estragole** | **-4.271** | **1.111** |
| **13** | **Citronellol** | **-4.471** | **0.722** |
| **14** | **(-)-Carvone** | **-4.372** | **1.565** |
| **15** | **Geraniol** | **-4.75** | **1.015** |
| **16** | **1-Decanol** | **-5.129** | **1.792** |
| **17** | **Anethole** | **-4.52** | **1.227** |
| **18** | **α-Copaene** | **-4.567** | **1.054** |
| **19** | **Caryophyllene** | **-4.709** | **1.921** |
| **20** | **(+)-Valencene** | **-4.836** | **1.486** |
| **#** | **ligand** | **-6.923** | **1.718** |

**Table S4.** Binding energies and RMSD of the 20 Compounds and ligand into the active pocket site of catalytic domain of GST (PDB: 3EIN).

| **NO** | **Compound** | **S_a_ kcal/mole** | **RMSD_Refine_b_** |
| --- | --- | --- | --- |
| **1** | **α-Pinene** | **-4.216** | **1.63** |
| **2** | **Sabinene** | **-4.509** | **1.727** |
| **3** | **α-Myrcene** | **-4.56** | **0.765** |
| **4** | **D-Limonene** | **-4.15** | **1.361** |
| **5** | **α-Ocimene** | **-4.523** | **1.716** |
| **6** | **γ-Terpinene** | **-4.454** | **0.987** |
| **7** | **1-Octanol** | **-4.355** | **1.337** |
| **8** | **Linalool** | **-4.407** | **1.571** |
| **9** | **Camphor** | **-3.492** | **0.631** |
| **10** | **(-)-Isomenthone** | **-4.208** | **1.451** |
| **11** | **Terpinen-4-ol** | **-3.969** | **1.844** |
| **12** | **Estragole** | **-4.273** | **1.78** |
| **13** | **Citronellol** | **-4.571** | **1.821** |
| **14** | **(-)-Carvone** | **-4.274** | **1.735** |
| **15** | **Geraniol** | **-5.861** | **0.868** |
| **16** | **1-Decanol** | **-4.599** | **1.457** |
| **17** | **Anethole** | **-4.07** | **1.837** |
| **18** | **α-Copaene** | **-4.456** | **0.966** |
| **19** | **Caryophyllene** | **-4.502** | **1.115** |
| **20** | **(+)-Valencene** | **-4.793** | **1.007** |
| # | **ligand** | **-5.945** | **1.405** |

**TableS5:** Receptor interactions and binding energies of Compound 15,16 and ligand into the active pocket site of catalytic domain of IL-1β (PDB: 6Y8M).

| **No.** | **Compound** | **S_a_ kcal/mole** | **RMSD_Refine_b_** | **Amino acid bond** | **Distance A֯** | **E (Kcal mol)** |
| --- | --- | --- | --- | --- | --- | --- |
| **15** | **Geraniol** | **-5.881** | **1.653** | **Asn 108 / H-donor** | **2.98** | **-0.8** |
|  |  |  |  | **Lys 109 / H-acceptor** | **3.08** | **-3.5** |
| **16** | **1-Decanol** | **-5.625** | **-4.625** | **GLN 149 / H-acceptor** | **3.39** | **-0.5** |
|  |  |  |  | **THR 147 / H-acceptor** | **3.25** | **-0.6** |
|  |  |  |  | **ARG 11 / H-acceptor** | **3.46** | **-0.60** |
| **#** | **ligand** | **-5.87** | **1.311** | **MET 148 / H-donor** | **3.04** | **-2.90** |
|  |  |  |  | **MET 148 / H-acceptor** | **3.08** | **-0.80** |
|  |  |  |  | **THR 147 / H-acceptor** | **2.74** | **-5.00** |
|  |  |  |  | **GLN 149 / H-acceptor** | **2.98** | **-3.50** |
|  |  |  |  | **ARG 11 / H-acceptor** | **3.15** | **-5.00** |
|  |  |  |  | **Arg 11 / Ionic** | **2.98** | **-4.60** |

^a^ S: the score of a compound placement inside the protein binding pocket.

^b^ RMSD_Refine: the root-mean-squared-deviation (RMSD) between the predicted pose and those of the crystal one (after and before refinement process, respectively).

**Table S6:** Receptor interactions and binding energies of Compound 8,15,16, and ligand into the active pocket site of catalytic domain of IL-6 (PDB: 1ALU).

| **No.** | **Compound** | **S_a_ kcal/mole** | **RMSD_Refine_b_** | **Amino acid bond** | **Distance A֯** | **E (Kcal mol)** |
| --- | --- | --- | --- | --- | --- | --- |
| **8** | **Linalool** | **-4.151** | **1.801** | **Arg 179 / H-acceptor** | **3.18** | **-2.3** |
|  |  |  |  | **Arg 179 / H-acceptor** | **3.23** | **-1.7** |
|  |  |  |  | **Arg 182 / H-acceptor** | **3.44** | **-0.5** |
| **15** | **Geraniol** | **-4.372** | **1.543** | **Arg 179 / H-acceptor** | **3.47** | **-0.9** |
|  |  |  |  | **Arg 182 / H-acceptor** | **3.46** | **-0.6** |
|  |  |  |  | **Arg 182 / H-acceptor** | **3.14** | **-2.6** |
| **16** | **1-Decanol** | **-4.401** | **1.106** | **Arg 179 / H-acceptor** | **3.25** | **-1.2** |
|  |  |  |  | **Arg 179 / H-acceptor** | **3.23** | **-0.6** |
|  |  |  |  | **Arg 182 / H-acceptor** | **3.03** | **-2** |
| **#** | **ligand** | **-4.191** | **1.758** | **Gln 175 / H-donor** | **2.78** | **-1.60** |
|  |  |  |  | **Arg 182 / H-acceptor** | **2.49** | **-3.50** |
|  |  |  |  | **Arg 182 /Ionic** | **3.06** | **-4.10** |
|  |  |  |  | **Arg 179 / H-acceptor** | **2.61** | **-4.30** |
|  |  |  |  | **Arg 179 /Ionic** | **2.51** | **-8.70** |

^a^ S: the score of a compound placement inside the protein binding pocket.

^b^ RMSD_Refine: the root-mean-squared-deviation (RMSD) between the predicted pose and those of the crystal one (after and before refinement process, respectively).

**TableS7:** Receptor interactions and binding energies of Compound 16 and ligand into the active pocket site of catalytic domain of TNF- α (PDB: 2AZ5).

| **No.** | **Compound** | **S_a_ kcal/mole** | **RMSD_Refine_b_** | **Amino acid bond** | **Distance A֯** | **E (Kcal mol)** |
| --- | --- | --- | --- | --- | --- | --- |
| **16** | **1-Decanol** | **-5.129** | **1.792** | **Gln 61 / H-donor** | **3.17** | **-0.7** |
| **#** | **ligand** | **-6.923** | **1.718** | **Gln 61 / H-donor** | **3.27** | **-0.7** |
|  |  |  |  | **Tyr 119/ pi H** | **4.12** | **-0.6** |

^a^ S: the score of a compound placement inside the protein binding pocket.

^b^ RMSD_Refine: the root-mean-squared-deviation (RMSD) between the predicted pose and those of the crystal one (after and before refinement process, respectively).

**Table S8:** Receptor interactions and binding energies of Compound 15 and ligand into the active pocket site of GST catalytic domain (PDB: 3EIN).

| **NO** | **Compound** | **S_a_ kcal/mole** | **RMSD_Refine_b_** | **Amino acid/ interaction** |
| --- | --- | --- | --- | --- |
| **15** | **Geraniol** | **-5.861** | **0.868** | **SER 66 / H-acceptor** |
|  |  |  |  | **Glu 65 / H-donor** |
| **#** | **ligand** | **-5.9455** | **1.4059** | **Glu 65 / H-donor** |
|  |  |  |  | **Ile 53 / H-donor** |
|  |  |  |  | **Arg 67 / H-acceptor** |
|  |  |  |  | **Ser 66 / H-acceptor** |
|  |  |  |  | **Arg 67 / Ionic** |
|  |  |  |  | **Glu 65 / Ionic** |

^a^ S: the score of a compound placement inside the protein binding pocket.

^b^ RMSD_Refine: the root-mean-squared-deviation (RMSD) between the predicted pose and those of the crystal one (after and before refinement process, respectively).


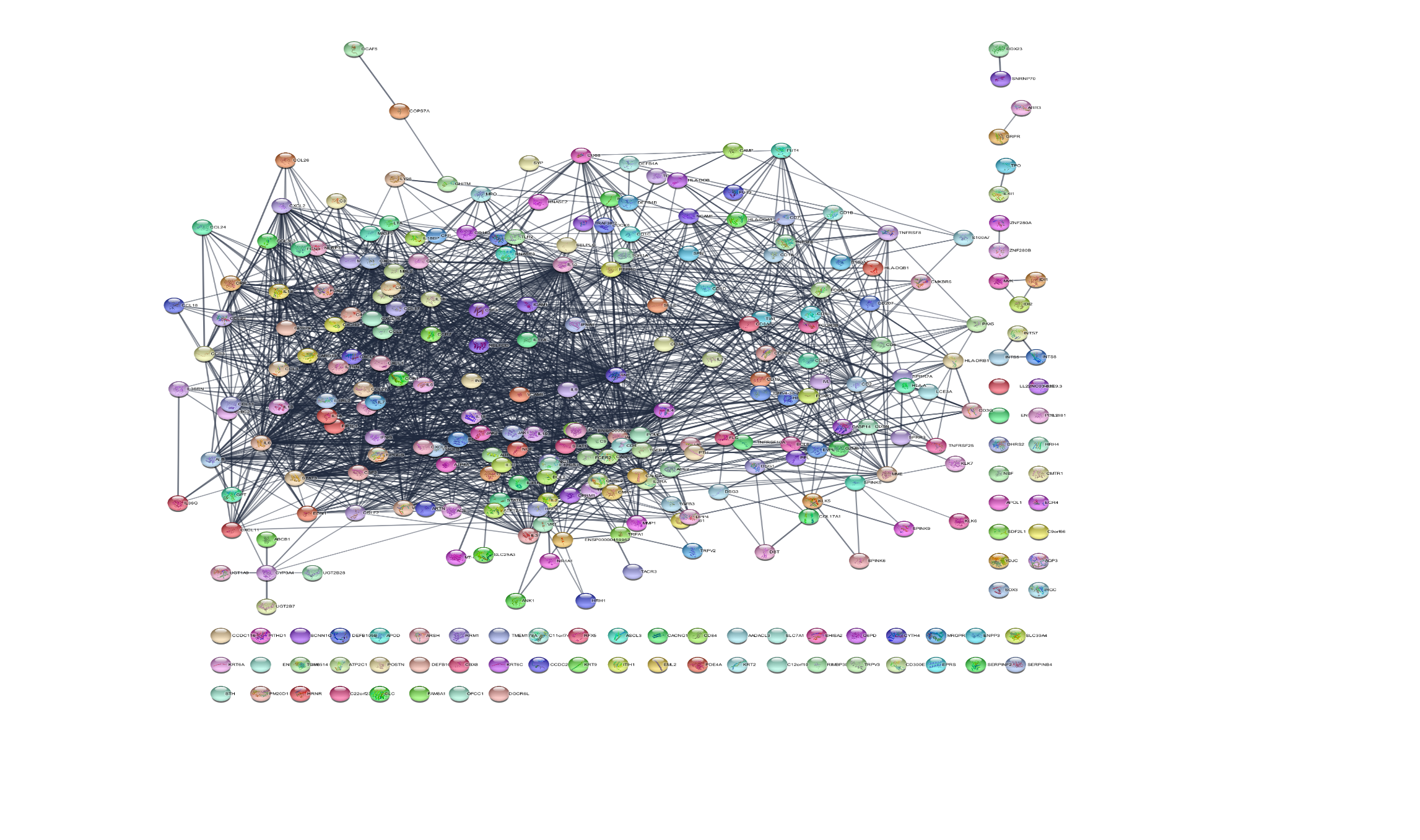


**Fig.S1**: Scabies network with organic layout, nodes represent protein targets, and the edges represent protein–protein interactions.


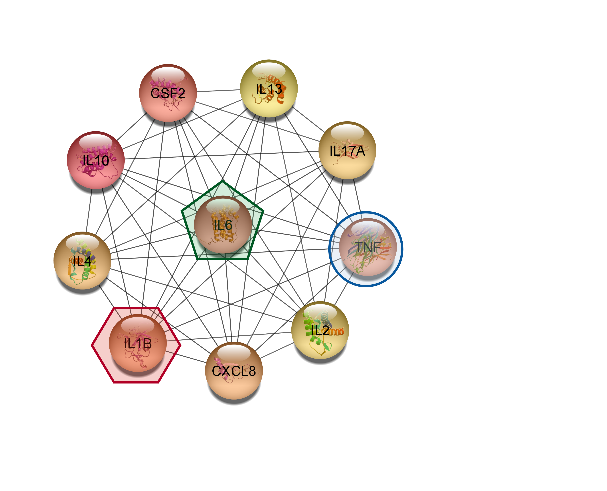

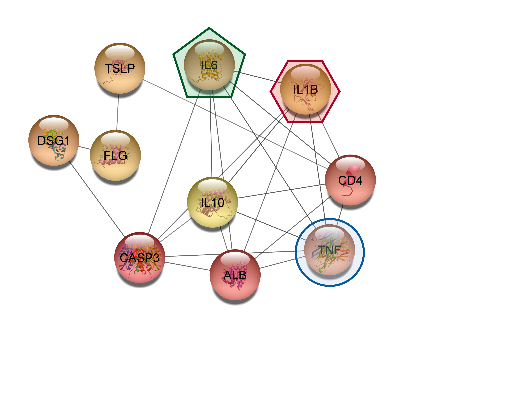


**Stress**

**MCC**

**MNC**


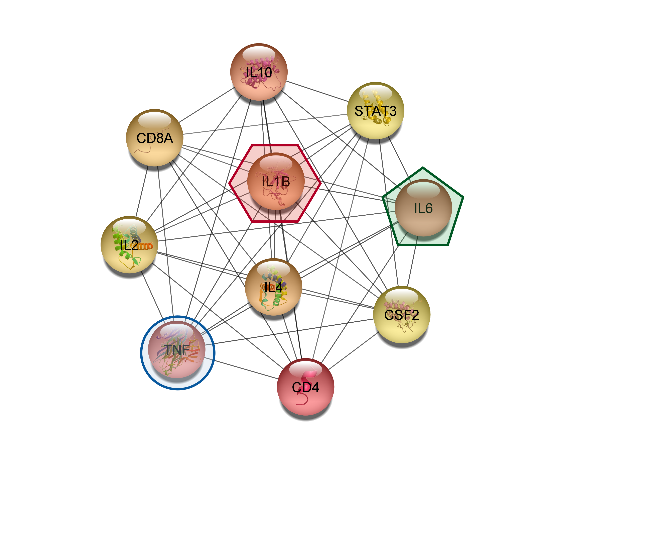


**Radiality**


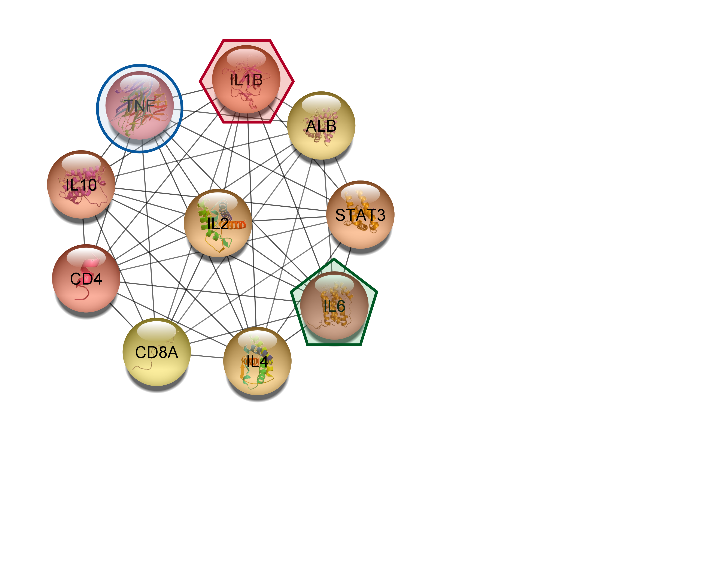


**EPC**


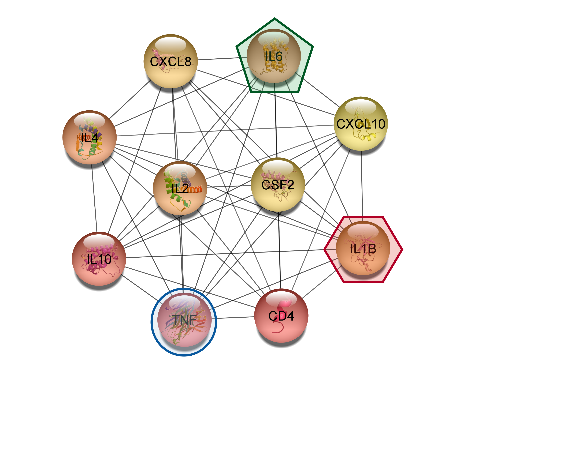


**Fig S2:** The occurrence of IL1B (red hexagon), TNF-α (blue circle) and IL6 (green pentagon) in analysis methods of cytoHubba.


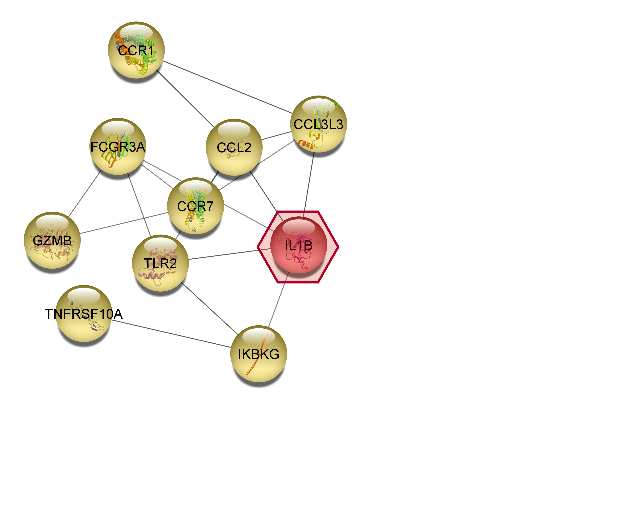

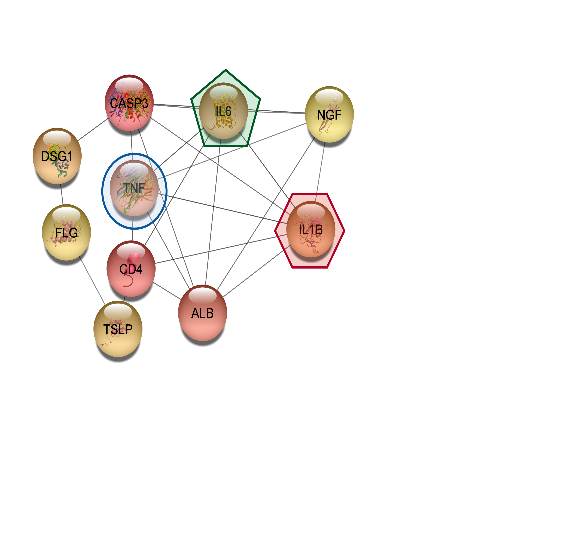


**EcCentricity**

**Betweenness**

**Closeness**


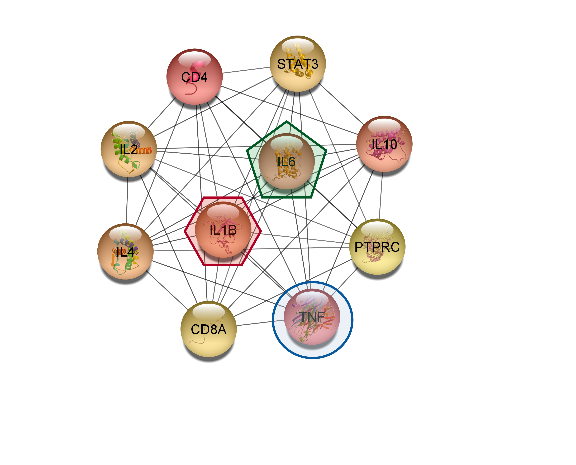


**Degree**

**Bottleneck**


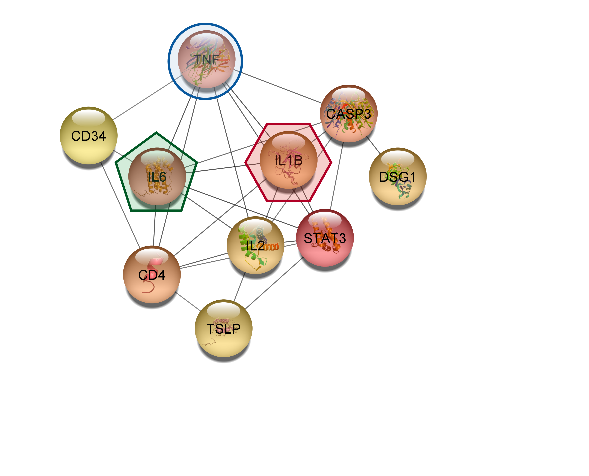

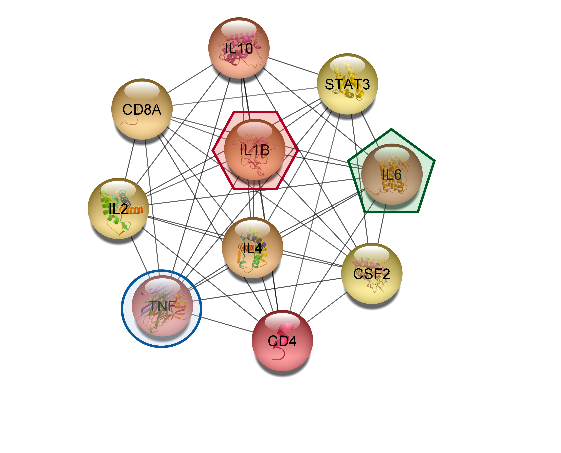


**Fig S3:** The occurrence of IL1B (red hexagon), TNF-α (blue circle) and IL6 (green pentagon) in analysis methods of cytoHubba
